# A quadriceps femoris motor pattern for efficient cycling

**DOI:** 10.1371/journal.pone.0282391

**Published:** 2023-03-16

**Authors:** Gernot O. Hering, Raphael Bertschinger, Jens Stepan

**Affiliations:** 1 Department of Sport and Health Science, University of Konstanz, Konstanz, Germany; 2 Department of Obstetrics and Gynecology, Paracelsus Medical University, Salzburg, Austria; Universita degli Studi di Milano, ITALY

## Abstract

In cycling, propulsion is generated by the muscles of the lower limbs and hips. After the first reports of pedal/crank force measurements in the late 1960s, it has been assumed that highly trained athletes have better power transfer to the pedals than recreational cyclists. However, motor patterns indicating higher levels of performance are unknown. To compare leg muscle activation between trained (3.5–4.2 W/kgbw) and highly trained (4.3–5.1 W/kgbw) athletes we applied electromyography, lactate, and bi-pedal/crank force measurements during a maximal power test, an individual lactate threshold test and a constant power test. We show that specific activation patterns of the rectus femoris (RF) and vastus lateralis (VL) impact on individual performance during high-intensity cycling. In highly trained cyclists, we found a strong activation of the RF during hip flexion. This results in reduced negative force in the fourth quadrant of the pedal cycle. Furthermore, we discovered that pre-activation of the RF during hip flexion reduces force loss at the top dead center (TDC) and can improve force development during subsequent leg extension. Finally, we found that a higher performance level is associated with earlier and more intense coactivation of the RF and VL. This quadriceps femoris recruitment pattern improves force transmission and maintains propulsion at the TDC of the pedal cycle. Our results demonstrate neuromuscular adaptations in cycling that can be utilized to optimize training interventions in sports and rehabilitation.

## Introduction

Force properties and the fiber structure of skeletal muscles are determined by the innervation paradigm of their afferent nerves [1]. This general principle in muscle physiology should also be key in prolonged, high-intensity cycling. Accordingly, there is strong evidence for differences in muscle morphology between high and low experienced cyclists [2–5]. World class cyclists have an exceptionally high economy of motion [6–8], which is usually determined by calculation of the gross efficiency (GE; S1 Fig), the ratio between mechanical output and energy expenditure [9]. Previous work also established an association between the proportion of type I fibers in leg muscles (e. g. VL) and GE in elite athletes [3, 9], which is very likely a result of the lower energy demands of type I fibers compared to type II fibers [10]. On the other hand, oxygen consumption can be reduced by more effective transmission of force to the pedals, known as index of force effectiveness (IFE) [11]. It is even conceivable that a certain pattern of activation improves IFE and leads to a higher proportion of type I fibers in the associated muscles through appropriate transformation stimuli. However, both scenarios increase GE and probably occur simultaneously under real world conditions. Despite this, there is currently no evidence to support the idea that improving IFE leads to improved GE, and no studies have confirmed a correlation between leg activation or coordination and performance. Studies in which subjects consciously altered their pedaling technique with [12], or without [13] force-feedback, found a slight increase in IFE which, however, was associated with an identical or higher oxygen consumption. However, no study has yet been published that identifies a specific recruitment pattern that is linked to cycling performance.

It is well accepted that muscle coordination can be described as redundant if pedal/crank force level is comparable [14–16]. Moreover, since the first reports of pedal/crank force measurements [17, 18], it has been assumed that top cyclists have a better power transmission to the pedals than recreational cyclists [19]. While most studies have found no performance-related differences in strength parameters [20, 21], a recent study reported higher IFE in professional cyclists compared to amateurs [22]. Methodological aspects can likely explain these contradictory results [22]. In previous studies, workloads were not adjusted to individual performance levels, so that inter-individual comparisons of muscular recruitment patterns may be biased [22].

In the current study, we used the established and validated performance marker MLSSw [23, 24] to correlate strength and electromyographical (EMG) measurements normalized to stable reference values with individual cycling performance. We hypothesize that certain leg muscle coordination patterns are associated with higher performance levels in cycling.

## Materials and methods

### Subjects

Fourteen competitive male cyclists, aged 21-40 years (26.3 ± 5 years) volunteered to participate in the study. Body high was 175-194 cm (183 ± 5.9 cm), body weight 64-92 kg (75.9 ± 6.9 kg). All subjects had at least 2 seasons of experience in road bike races accredited by a national/international cycling federation (11 ± 7 years). One subject (9) was an elite cyclist at Tour de France level. Among the remaining participants was one ambitious recreational athlete, one semi-professional athlete, and eleven athletes holding amateur licenses of German category A-C. Each participant gave written, informed consent after being provided a detailed description of the study requirements and procedures. The experimental procedures were approved by the University of Konstanz Institutional Review Board and were performed in accordance with the ethical standards of the government of Baden Württemberg. All subjects were healthy and were instructed to avoid heavy exercise and to maintain a normal diet at least 2 days before testing.

### Definition of group A (highly trained) and group B (trained)

“Trained” and “highly trained” are arbitrary terms whose interpretation is subjective and sport-specific. Here, the terms are objectified based on the maximal lactate steady state (MLSS). Workload at physiological thresholds such as MLSS, onset of blood lactate accumulation (OBLA) and ventilation threshold (VT) are recognized, objective, and validated performance markers that can be used to accurately evaluate the individual performance level [24–27]. The ILT-test used here provides a precise (7-10W), reproducible and valid measurement of the steep capillary lactate increase for MLSSw determination [24]. On this basis, subjects were assigned to group A, (highly trained, mean MLSSw/kg 4.51 W/kgbw (max = 5.1, min = 4.3), MLSSw = 339 W (max = 384, min = 290), BW = 75.4 kg, BH = 183.3 cm) or group B (trained, mean MLSSw/kg 3.94 W/kgbw (max = 4.2, min = 3.5), MLSSw = 299 W (max = 327, min = 267), BW = 76.4 kg, BH = 182.7 cm) according to their MLSSw/kgbw (both groups n =7). Although the MLSSw as well as the MLSSw/kg differ in "professional" and "elite" cyclists [26], here, the MLSSw related to body weight seems to be the most appropriate parameter on the topic of pedaling economy and muscle coordination.

### Experimental setup

All participants completed two tests on separate days on a bicycle ergometer. The first test was an “individual lactate threshold” (ILT)-test to determine maximal lactate steady state workload (MLSSw) [24]. The second test comprised three, 4 min intervals at constant workload (CP-test), which was individually set according to ILT-test results. On both days, we performed EMG recordings of four lower limb muscles of the right leg. In order to conduct inter-subject comparisons, all data were normalized to crank force maxima. Therefore, subjects performed a maximal power (MP)-test prior to ILT- and CP-tests (for details see next section). Before MP-tests, all participants performed a 10 min warm-up at an intensity of 2 W/kgbw. MP-tests were followed by an active recovery period of 10 minutes at 1.5 W/kgbw. Cadence was self-selected by the participants during the warm-up session and used as reference for all following experiments. If cadence deviated by more than 3 rpm, athletes were advised to adapt their pedaling speed. Subjects were instructed to keep the same posture (hold on to the upper section of the handlebar) on the ergometer throughout all experiments, because previous research indicated EMG changes after upper body movements [28]. The cycling position on the bicycle ergometer was adjusted according to the dimensions of the athlete’s personal bicycle and kept constant on all test days. Due to methodological reasons we decided to limit EMG recordings to four muscles: (i) RF, hip flexor and knee extensor; (ii) VL, largest single-joint knee extensor; (iii) semitendinosus (ST), hip extensor and knee flexor; (iv) tibialis anterior (TA), strongest dorsiflexor of the foot.

### Test protocols

#### Maximal Power (MP)-test - data normalization

EMG data normalization is crucial for reliable and objective inter-subject as well as inter-muscle comparisons [29]. Thus, MP-tests were executed for force- and EMG-data normalization, and also for normalizing data to body posture. This procedure was verified in several pilot experiments. MP-tests started with a resistance of 60 W, which was increased by 40 W every 2 seconds until exhaustion.

#### Individual Lactate Threshold (ILT)-test

The lactate threshold and the MLSSw were assessed by the “individual lactate threshold test” (ILT-test) as previously described (S2 Fig) [24]. [La^-^] was measured with Arkray Lactate Pro^®^ LT-1710, sampling size 5μl, measuring time 60s, coefficient of variability (CV) = 3% [30].

#### Constant Power (CP)-test

CP-tests comprised a set of three intervals at constant workload at 90%, 100% and 110% of the previously determined MLSSw. The intervals were randomized between subjects in order to minimize effects of fatigue when analyzing data. In order to avoid high levels of fatigue, interval length was set to 4 min and exercise bouts were separated by active, 5 min recovery sessions at an intensity of 1,5 W/kgbw.

### Cycling ergometer and force measurement device

Experiments were conducted on a modified bicycle ergometer (Lode Excalibur, Lode B. V., Groningen, The Netherlands) equipped with a Powertec bi-pedal/crank force measurement device (O-tec, Bensheim, Germany) [31], with custom made optical rotatory distance registration. In order to synchronize all mechanical as well as EMG data to a specific crank angle, an optical encoder (E3 Optical Kit Encoder, US Digital, Washington, USA) with a resolution of 0.45° angular degrees (800 data points per crank cycle) was mounted between the bottom bracket and the cranks of the bicycle ergometer (S3 Fig). The system was configured to a zero-degree-position of the right crank arm pointing in a vertical upright direction, called top dead center (TDC) [20]. The crank length was fixed at 175 mm. To maintain a high accuracy of the force measurement device, the system was calibrated at least once a day and after changing the pedals.

### EMG-recordings

EMG data of RF, VL, ST and TA of the right leg were collected during all tests by means of custom-developed, bipolar Ag-AgCl surface electrodes (S3 Fig). One pair of electrodes consisted of a detection and a reference electrode. The detection electrode was equipped with a built-in preamplifier [32] that reinforced the incoming signal 20-fold. EMG-signals were high- and low-pass filtered with a bandwidth of 20-500 Hz. Subsequently EMGs were amplified according to their maximal amplitude 250/500-fold, thus, directing a 5000/10000-fold amplified signal to the AD-converter.

### Electrode application

At contact sites between the skin and the EMG electrode bars, the skin was shaved, cleaned with alcohol and roughened with sandpaper to reduce resistance and increase conductivity between the electrode and the skin. The detecting electrode was placed onto the muscle belly, distally between the innervation zone (motor point) and the tendon origin. The electrode bars were oriented orthogonally to the muscle fibers for the VL, ST, and TA. For the RF, electrode bars were oriented perpendicular to the longitudinal axis of the muscle. The reference electrode was attached in parallel to the detecting electrode at a distance of about 1.5 cm. S3 Fig shows the electrode configuration for each of the lower limb muscles. Before recordings started, EMG signals were visually checked for artefacts with an oscilloscope. The electrode design and attachment method were executed as previously described [29] and were continuously refined. Only the two electrode bars were in contact with the abraded skin and caused a slight skin discoloration (S3 Fig). Thus, electrodes could be placed in the identical position in the same transverse and sagittal plane on different days.

### Data processing

Analogue signals were registered using a 12-bit AD-converter with two 64 KB (32 KSamples) switch buffers (ISC16 RC Electronics Santa Barbara, USA). The analogue voltage range encompassed ± 10 V. Data of all 16 channels were collected at a sampling rate of 2 kHz. While one buffer recorded data, the other read the previously captured data. This resulted in a storage interval of 1 second for both alternating buffers of the AD-converter. When data acquisition of buffer A was finished, data acquisition switch to buffer B and the data of buffer A were saved in a RAM-array. This procedure was repeated 5 times (=5 seconds). Before the next data acquisition cycle begins, the data stored in the 5s RAM-array were analyzed and send to the screen for graphical and quantitative presentation (see also S4 Fig).

Two methods were used to calculate force, time and EMG parameters in CP- and ILT-tests. First, the mean values were calculated synchronously (time-based) over 180°/360° for each crank cycle. Second, data from two pedal cycles (1600 data points), were stationary synchronized using the optical decoder and averaged over a defined test period (dataset averaging, see below). Stationary synchronization is the allocation of every data point to a specific angular degree of the pedal cycle. The high-resolution encoder produced square-wave signals on two analog channels (400 per channel = 800 in total), to which the software assigned synchronized (time-based) force or EMG values. Another analog channel provided the square wave signal for the TDC.

The resolution of this synchronization method was 0.45° (360° divided by 800 data points). The amount of completed crank cycles within a 5 second interval, defined the amount (n) of averaged data. For example, at an average cadence of 94 rpm, a cyclist performed 7 complete crank revolutions in a 5 second interval. In this case, a 4 min CP-test contained 336 crank cycles and yielded 48, five-second data packages. After data averaging (here, averaging of 2 complete crank cycles = 720°) we thus obtained 3 data sets per 5 seconds, which corresponds to 144 averaged datasets in a 4 min CP-test interval.

Averaged data sets were corrected by a factor (fcor = mean of all data set maxima ÷ maximum of the averaged data set) that incorporated differences in amplitude latencies. This preserved the correct maxima of signal amplitudes resulting in a stable stationary pattern after approximately 40-50 cycles of averaging.

5-second data packages were manually/visually checked for errors and marked for further analysis. Subsequent normalization and calculation of parameters was performed using custom written software. To avoid signal interference, EMG and force maxima used for individual normalization were visualized and quantified at 5-second intervals. We used absolute maxima as reference values, since calculation of integral windows, means, or maximum averages showed no advantage.

Onset and termination of innervation were determined automatically for the time-synchronized data using a self-developed software algorithm. For averaged data sets, we manually identified the beginning and end of muscle activity. Definitions of all parameters are shown in S1 Fig.

### Data analysis and statistics

Statistica (Statistica 6.0, StatSoft, Tulsa, USA) and custom written software was used for data analysis. Data were analyzed by two-tailed paired and unpaired *t*-tests as appropriate. F-tests were used to test for equality of variances. Correlations between variables were assessed using Pearson’s correlation coefficient (*r*). Data are expressed as a mean value ± SD unless indicated. *P* values of ≤ 0.05 are considered significant and denoted with *, ≤ 0.01 with **, and ≤ 0.001 with ***.

## Results

### Time synchronized and averaged data link quadriceps femoris activity pattern to individual cycling performance in CP- tests

**Fig 1:**
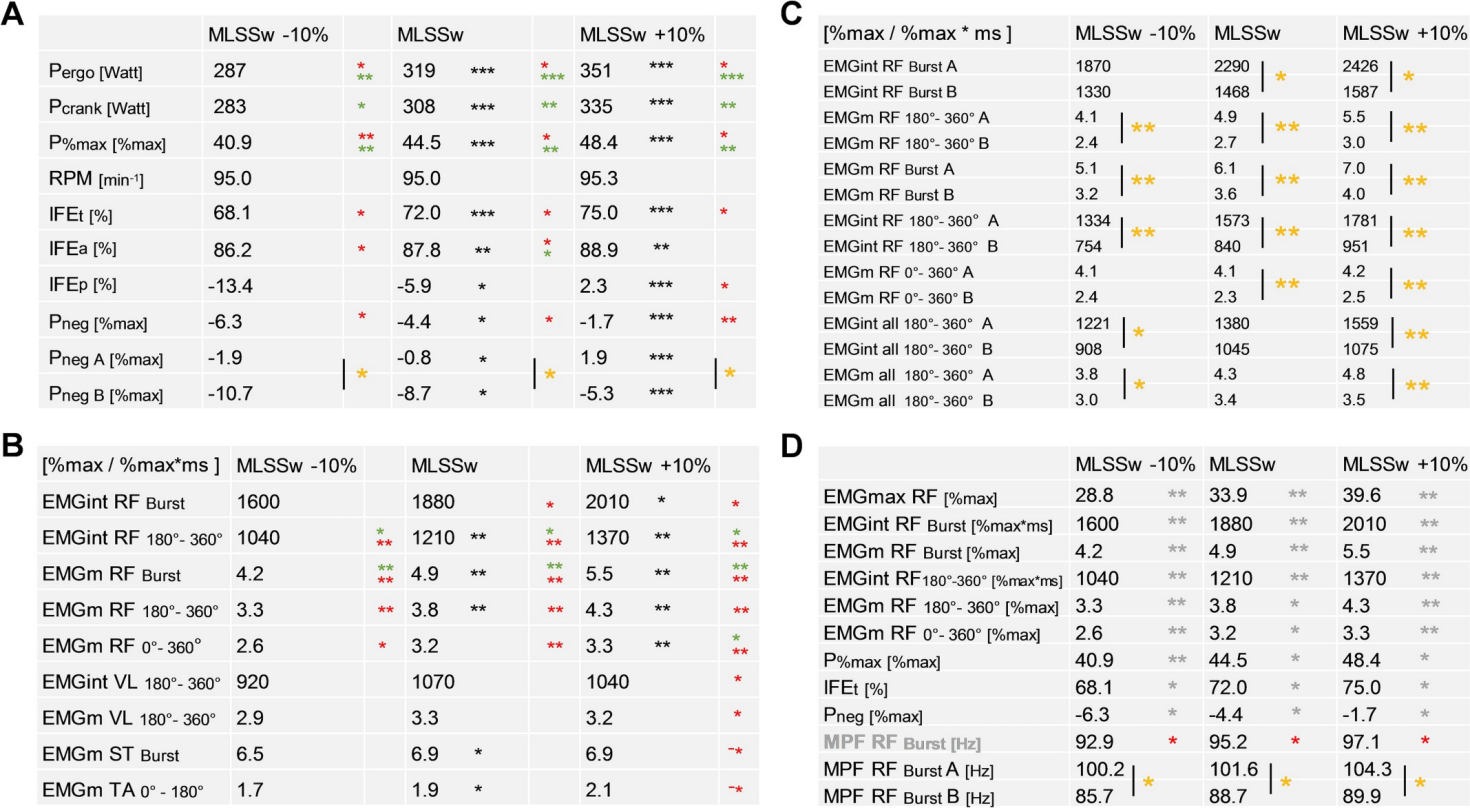
Time synchronized force and EMG data in CP-tests. Results are shown for MLSSw - 10%, MLSSw, and MLSSw + 10% **(A-D)**. **(A)** Correlation of mechanical parameters to MLSSw and MLSSw/kg (n = 14). Except for cadence (RPM), all parameters differ between workloads. Negative force is lower in group A. **(B)** Correlation of muscle activity to MLSSw and MLSSw/kg (n = 14). Only RF activity consistently differs between workloads and correlates to MLSSw and MLSSw/kg. The only exception is the VL at MLSSw + 10%. Note the negative correlation for ST and TA to MLSSw/kg at the same workload. **(C)** RF activity and correlation to MLSSw and MLSSw/kg at different workloads for group A and B (both groups n = 7). RF activity but also mean EMGs of all 4 muscles were consistently increased especially in the posterior part of the pedal cycle in group A compared to group B. **(D)** Correlation of frequency parameters (MPF) to IFEt, P%max, Pneg and EMG RF (n = 14). Note the pronounced MPF of RF in group A, which likely increases the quantitative amount of neural drive and, thus, the contractile force. P%max and Pneg are shown as mean of both cranks. IFE(t,a,p) is shown as mean of the right crank. For detailed description of data analysis and parameter definitions see Methods and S1 Fig. Black asterisks (*): two-tailed paired t-tests **(A,B)**, yellow asterisks (*): two-tailed unpaired t-tests **(A,C,D)**, red asterisks (*): correlation to MLSSw/kg **(A,B,D)**, green asterisks (*): correlation to MLSSw **(A,B)**, grey asterisks (*): correlation of MPF RF Burst to mechanical parameters and quantitative RF activity **(D)**. Shown is mean. *P < 0.05, **P < 0.01, ***P < 0.001.

First, we examined the consistency of our force measurements. Bi-pedal crank power (Pcrankt, P%max) increased proportionally with ergometer power (Pergo) at constant cadence (RPM) in CP-tests (Fig 1A). Remarkably, increasing Pergo was accompanied by increasing mechanical effectiveness (IFE(t,a,p), and decreasing negative power (Pneg, Fig 1A). Moreover, IFE(t,a) and Pneg correlated with MLSSw/kg, and Pneg was lower in group A than in group B (Fig 1A).

Next, we investigated recruitment patterns in response to workload variations calculated for single pedal revolutions. Here, we recorded clear differences between workload levels only for RF activity (Fig 1B). Furthermore, we found a strong correlation between RF activity in the posterior half (180°-360°) of the pedal cycle and MLSSw/kg in all three tests (Fig 1B). At the MLSSw +10%, there was a positive correlation between VL and MLSSw/kg in the posterior half of the pedal cycle, but also a negative correlation for ST/TA and MLSSw/kg in the anterior half of the pedal cycle (Fig 1B).

To investigate the influence of performance level on muscle activity, we next compared groups A and B. In all three tests, group A athletes had consistently higher RF activity within the posterior half of the pedal cycle than group B athletes. Moreover, EMG activity of all muscles (EMG all 180°-360°) was significantly increased in group A, suggesting that these athletes innervate a comparatively larger part of their muscle mass within the posterior half of the pedal cycle (Fig 1C). This assumption was supported by the correlation between RF power spectrum (MPF, for physiological interpretation see [33, 34]) and individual performance (MLSSw/kg), and by the correlation between MPF RF and RF excitation intensity (EMGint RF/EMGm RF) (Fig 1D). Finally, the correlation between MPF RF and RF excitation peaks (EMGmax RF) in highly trained individuals indicated the recruitment of larger motor units resulting in reduced Pneg, higher relative power (P%max) and higher mechanical effectiveness (IFEt) (Fig 1D).

**Fig 2:**
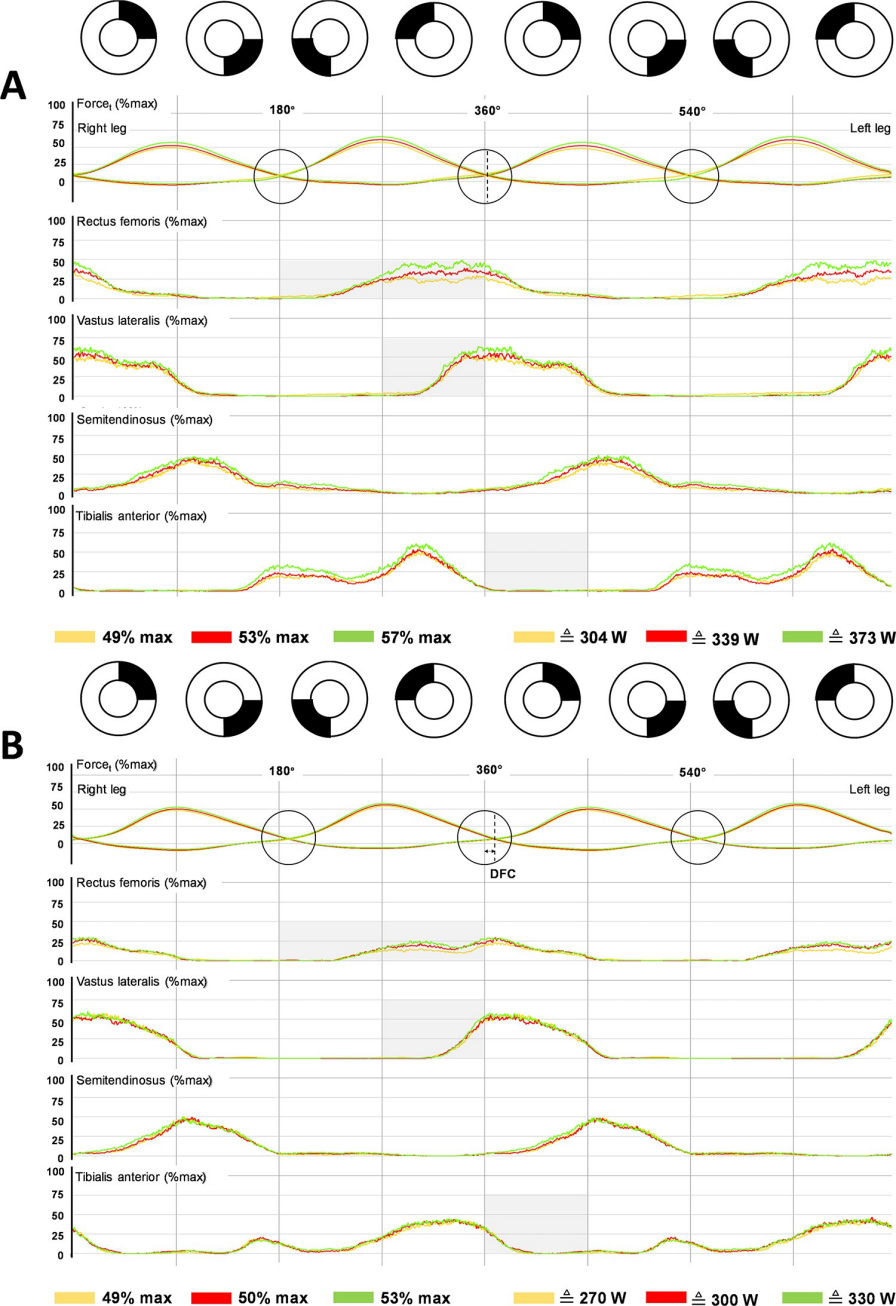
Group comparisons of recruitment patterns of lower limbs muscles at the MLSSw in CP-test. **(A, B)** Averaged bi-pedal crank force and EMG data across two pedal cycles (0–720°) at MLSSw–10% (yellow), MLSSw (red) and MLSSw + 10% (green) for group A **(A)** and group B **(B)** (both n = 7). Group comparisons reveal differences in force development, negative force level and recruitment patterns for RF, VL and TA (see gray shaded areas). Note also the vertical crossing points of the tangential force (Ft) and the delay at the TDC (Delay Ft crossing = DFC) at 180°/360°/540° in marked circles.

**Fig 3:**
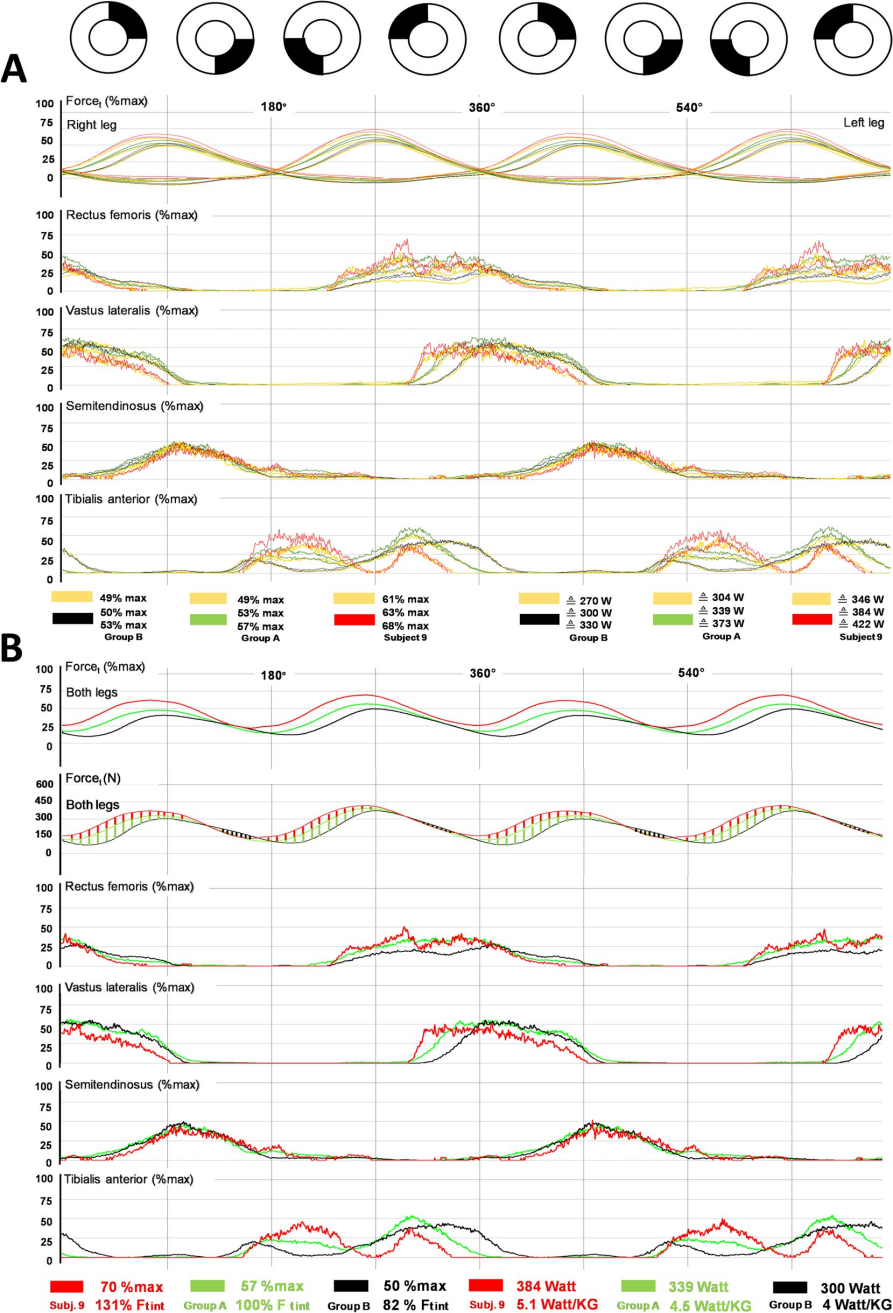
Group comparisons of recruitment patterns and propulsive force around MLSSw in CP- test. (**A)** Averaged bi-pedal crank force and EMG data across two pedal cycles (0–720°) of group A (green), B (black) and subject 9 (red). In CP-tests we recorded an earlier rise of EMG activity in Q3/Q4 and force development in Q1 in athletes with a higher MLSSw/kgbw. Note the more pronounced increase of RF and VL activity in group A and subject 9 at the transition from MLSSw to MLSSw +10%. **(B)** Averaged total propulsive force (Ft r+l) and EMG data across two pedal cycles (0–720°) at the MLSSw for group A (green, n = 7), group B (black, n = 7) and subject 9 (red). **(B, second line)** depicts curves for the absolute propulsive force. The early and strong rise of RF/VL activity prevents the loss of propulsive force (Ft r+l) at the TDC and increases the force development in Q1 (shaded red and green areas). Ftint of group A is 18% larger compared to group B. Subject 9 even had a 49% higher Ftint than group B **(B, top row)**.

Just like the time synchronized data (Fig 1), averaged data (S1 Fig) confirmed the considerable redundancy of muscle recruitment patterns during the apparently uniform movement of pedaling (Figs 2, 3, S5 and S6 Figs). The RF and VL systematically determined force development, mainly in the first and fourth quadrant (Q1/Q4) of the pedal cycle. The ST was mainly active in Q1/Q2, while the TA was mainly active in Q3/Q4 (Fig 3A and 3B). RF and VL activation intensity and timing appeared to be individual performance criteria, revealed by the strong correlation of their activity in Q1/Q4 with MLSSw and MLSSw/kg (S6 Fig).

**Fig 4:**
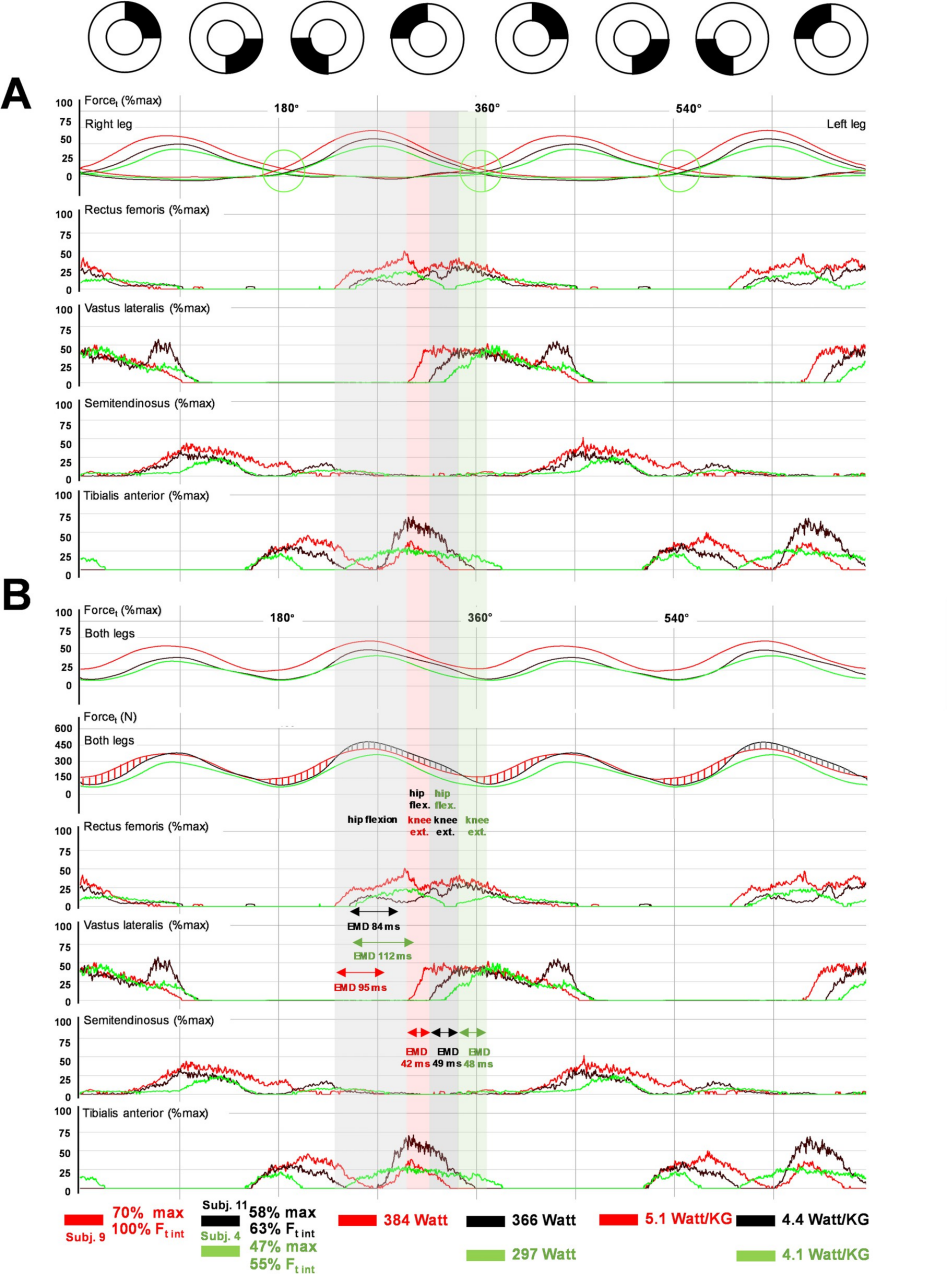
Examples of RF/VL recruitment pattern and force development at the TDC for subjects at different performance levels in CP-tests. (A) Averaged bi-pedal crank force and EMG data across two pedal cycles (0–720°) for athletes with a similar, high MLSSw (subject 9, red) and subject 11, black) and a lower MLSSw (subject 4, green). Note the vertical crossing points of the tangential force (Ft) and the delay at the TDC (Delay Ft crossing = DFC) at 180°/360°/540°. Although the DFCs of subjects 9 and 11 hardly differ (1.4°/2.4°), force development of subject 11 is clearly delayed in Q1. The high DFC of subject 4 (8.6°) results in an even more pronounced delay of force development. Note the double burst of the RF in subject 4. (B) Averaged total propulsive force (Ft r+l) and EMG data across two pedal cycles (0–720°) for subject 9 (red), 11 (black) and 4 (green). The relative force integral (Ftint) of subject 9 is larger than that of subject 11 (37%) and 4 (45%). Thus, with this pattern of recruitment, 37% and 45% more relative power is available for propulsion in subject 9, respectively (B, top row). (B, second line) depicts curves for the absolute propulsive force. At similar workloads (384W/366W), subject 9 has the highest force development in Q1 (red shaded areas). In contrast, subject 11 has the highest force development in Q2, during RF activity that causes hip flexion at the contralateral side of the pedal cycle (247°-354°, black shaded areas). Therefore, force distribution over a pedal revolution is more uniform in subject 9. Note also the different electromechanical delays (EMDs) and innervation onsets of the RF/VL of subjects 9, 11 and 4. Subject 9 innervates the RF from hip flexion to knee extension earlier and more intensely. Furthermore, the onset of innervation of the VL is later in VP 11 and, to an even higher extent, also in VP4. This interruption of muscle activity clearly shifts the onset of recruitment of knee extensors towards Q1 (see also Fig 6).

**Fig 5:**
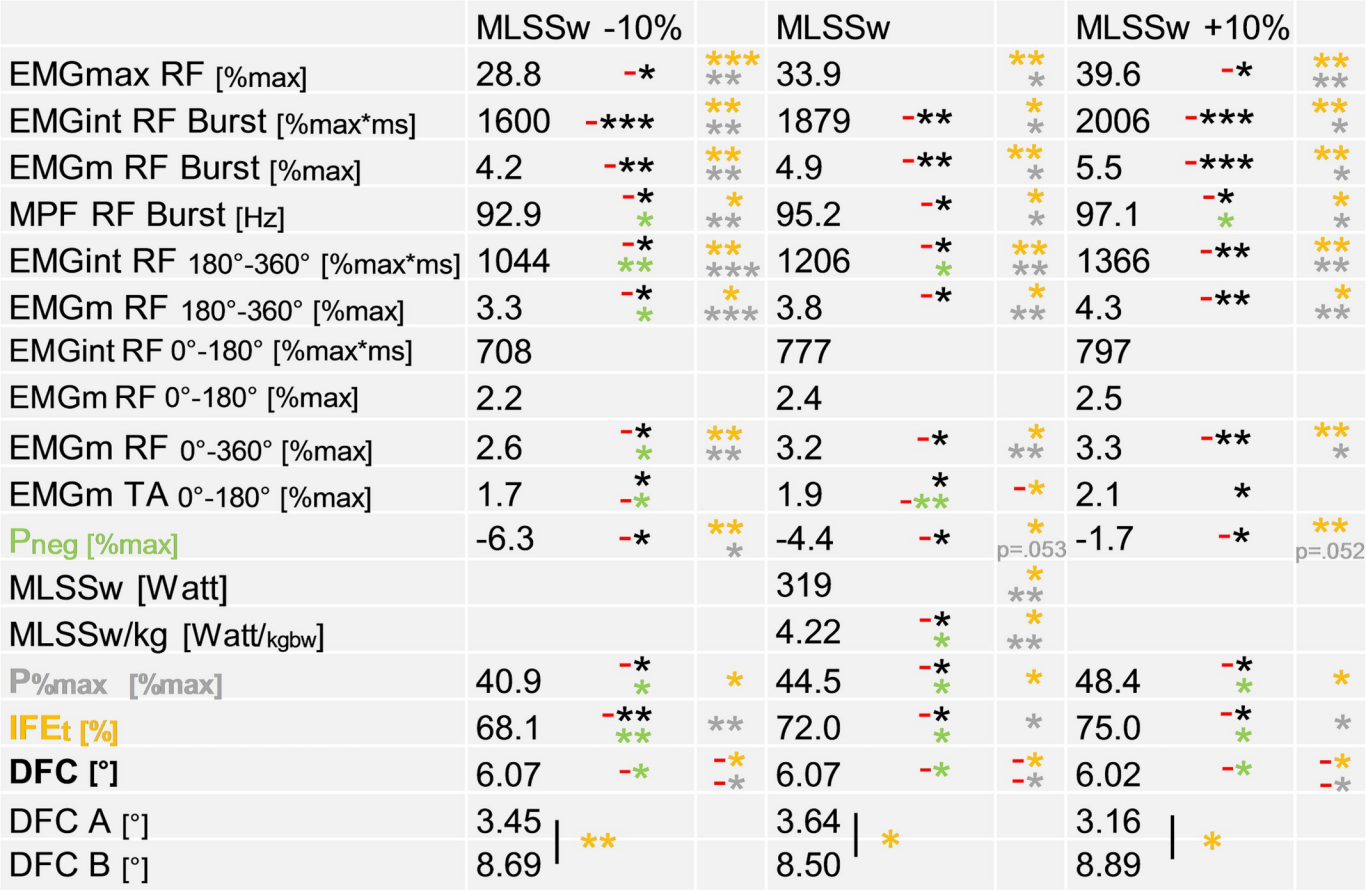
Correlations of force and EMG parameters relevant for coordination and efficient force production in CP-tests (n=14). These data show that the retrievable power at the MLSSw (P%max), the negative power (Pneg) and the mechanical effectiveness (IFEt) is determined by a strong and early activation of the RF in Q3/Q4. DFC, IFEt, Pneg and P%max are correlated to MLSSw/kgbw and are highly influenced by RF activity. Our data suggests that TA activation in the first half of the pedal cycle (Q1, see Figs 2 and 3) impedes cycling performance. Note that DFC is significantly reduced in group A and is inversely correlated to EMG and force/power parameters. Data were calculated for each pedal cycle. P%max and Pneg are shown as mean of both cranks. IFEt is shown as mean of the right crank. For detailed description of data analysis and parameter definitions see Methods and S1 Fig. Black asterisks (*): correlation to DFC, grey asterisks (*): correlation to P%max, yellow asterisks (*): correlation to IFEt, green asterisks (*): correlation to Pneg. Shown is mean. *P < 0.05, **P < 0.01, ***P < 0.001.

To better understand the relationship between performance level and muscle activity patterns we next focused on group comparisons. We found an earlier and stronger RF/VL activation in group A (Figs 2 and 3), resulting in lower negative force (Fneg) and faster increase of propulsive force (Ft) at the contralateral crank. Hence, crossing of left and right tangential forces (Ft) occurred earlier and, thus, closer to the TDC (180°, 360°, 540°) (Figs 2 and 3). The delay between the TDC and crossing of left and right Ft (DFC) was negligible in group A but pronounced in group B (Figs 2, 3 and 5). The minor DFC in group A and especially in test subject 9 (Figs 2–4) obviously reduced loss of propulsion at the TDC (Figs 3 and 4), and enables a steeper rise of Ft in Q1 (Fig 3). The relationship between DFC, RF activity, IFE and Fneg/Pneg is shown in Fig 5. TA activity in the anterior half of the pedal cycle (mainly in Q1) seemed to constrain cycling performance (Figs 1B and 5).

The key role of specific muscle coordination strategies in cycling is illustrated by in-depth analysis of four datasets (S6 Fig). Subject 9, a top-level cyclist, mainly differed from the other athletes by early and strong activation of the RF and VL in the second half of the pedal cycle, especially in Q4 (Figs 3–5, S6 Fig). This allowed this athlete to generate comparatively higher power in Q1, while minimizing or avoiding Fneg in the second half of the pedal cycle. The VL supported this early RF activation, whereas the TA was progressively activated in Q3 and to a lesser extent also in Q4. In contrast, less powerful athletes had reduced RF activation in favor of VL activation in the second half of the pedal cycle (S6 Fig; subjects 10,11,12).

**Fig 6:**
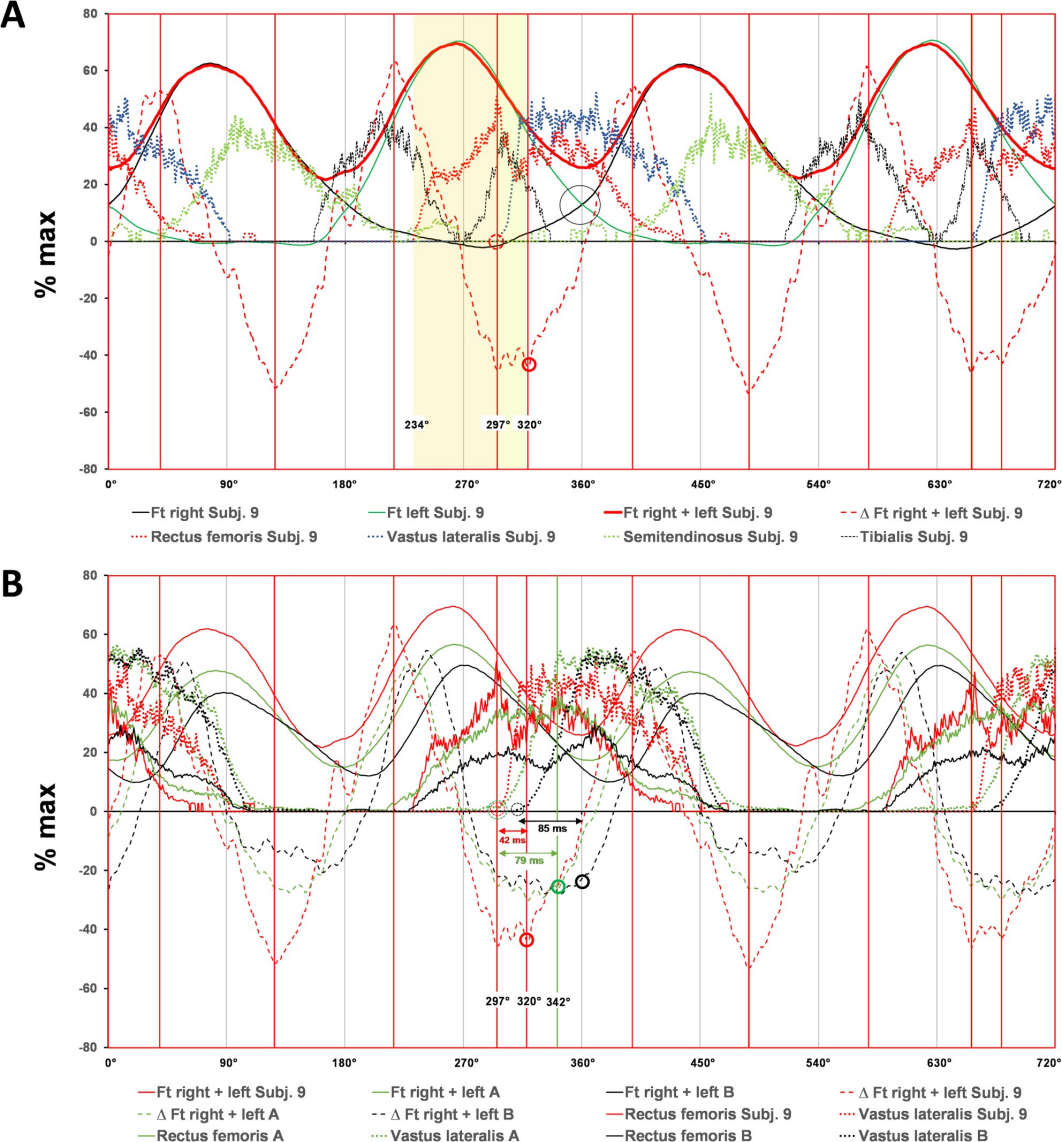
Recruitment pattern of RF/VL at the transition from hip flexion to knee extension in CP-tests. **(A, B)** Bi-pedal crank force (Ft right and Ft left), total propulsive force (Ft r+l), rate of force development (ΔFt), and averaged EMG across two pedal cycles at the MLSSw for subject 9 (red) **(A)** and for subject 9 vs highly trained (group A) and trained (group B) athletes **(B)**. Vertical lines and circles (red = subject 9, green = group A, grey/black = group B) show reversal points of ΔFt (begin of force development during knee extension). Broken circles (red = subject 9, green = group A, black = group B) indicate the beginning of VL activation. Note the delayed VL activation (297°-320°) after termination of RF activity for hip flexion (234°-297°) in Q4 **(A)**. Here, the RF likely operates together with the VL as a knee extensor. After onset of activation, the VL/RF-induced change in ΔFt (electromechanical delay, EMD) starts after 42 ms in subject 9, after 79 ms in group A and after 85ms in group B **(B)**. A major difference between group A/subject 9 and group B is the more intense RF activation and the rapid onset of VL activity. Here, the pretension of the RF during hip flexion and subsequent strong and fast knee extension together with the other thigh extensors is the characteristic activation pattern of highly endurance trained cyclists **(B)**. For detailed description of data analysis and parameter definitions see Methods and S1 Fig.

The detailed RF and VL recruitment patterns at the TDC are illustrated in Fig 6. The initial strong RF activation in subject 9 is consistent with its function as a hip flexor (Fig 6A). After a short decline in activity, the RF was activated again together with the VL. This switch of RF function, from hip flexion to knee extension, seems to be important for activation of the VL and probably all quadriceps extensors with little or no delay (Fig 6A). The comparison of groups A and B revealed two important differences. Athletes in group B had lower RF activity and a twofold longer EMG force delay (EMD) of the VL (Fig 6B). The EMD describes the delay between onset of muscle activation and onset of Δ Ft (Fig 6). Strong RF activation before knee extensor activation resulted in higher total propulsive force (Ft) in Group A and, most strikingly, in subject 9 (Fig 6). This activity pattern is consistent with correlations between RF/VL activation and relative crank power (P%max), Pneg, MLSSw and MLSSw/kg (Figs 1B, 1D and 5). Thus, a pronounced RF activation offers three key advantages for prolonged high-intensity cycling: A reduced negative power and reduced loss of propulsive force at the TDC and an early activation of the knee extensors to can generate higher propulsive force in Q1. Importantly, semitendinosus recruitment patterns exhibited no group differences.

Taken together, individual muscle coordination and force variation within a crank cycle can be captured very precisely by our data acquisition and analysis strategy. Both time synchronized data and stationary synchronized data (averaged data) demonstrate a clear relationship between level/timing of RF/VL activation and individual performance (MLSSw/kg) in cycling.

### Modified EMG-crank force relationship during MP- tests

In ramp tests with continuously increasing resistance, subjects were not given any instructions on pedaling technique, but were advised to remain in a seated position, holding on to the upper section of the handlebar, and maintain a constant cadence.

**Fig 7:**
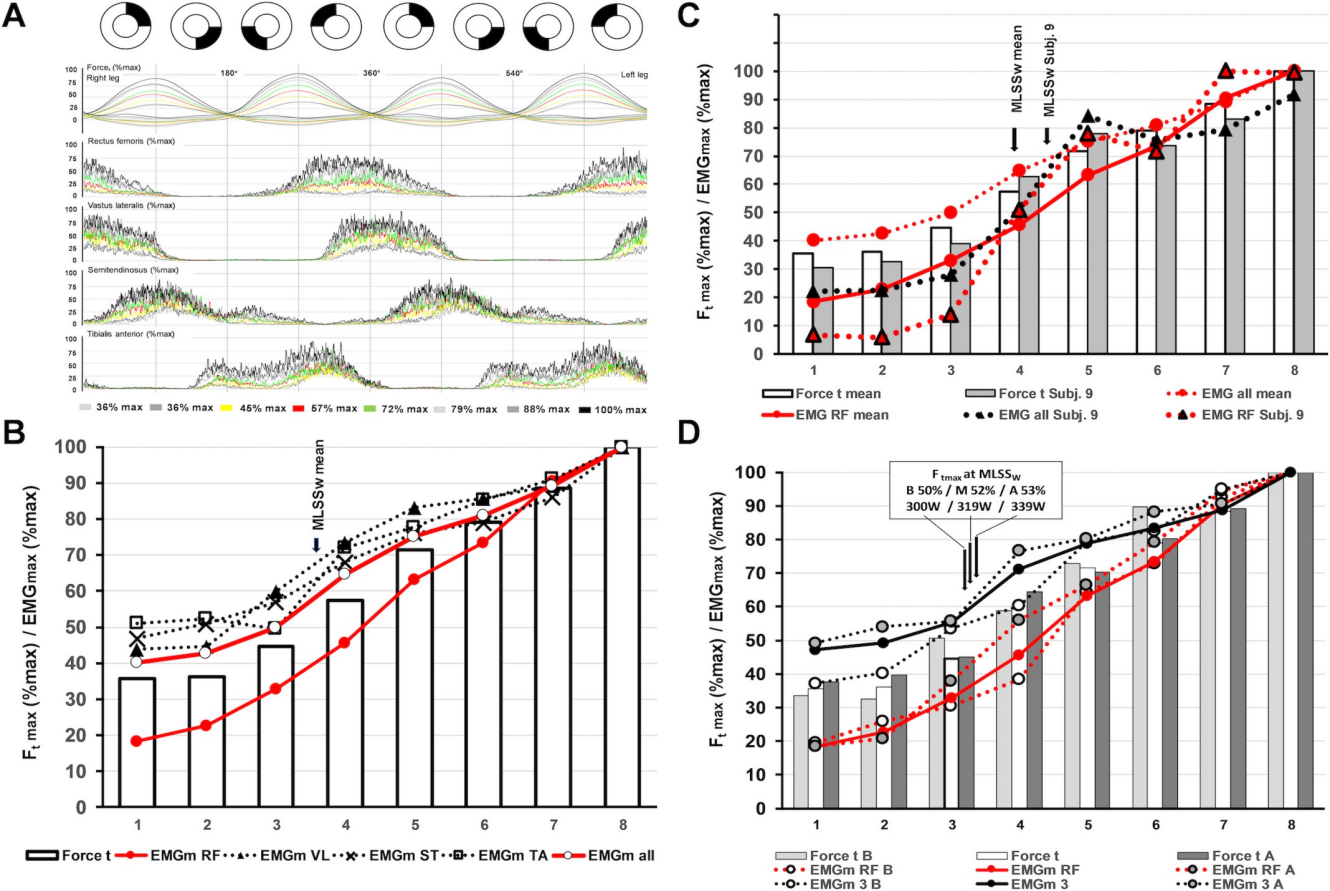
Summary of the force/EMG pattern during MP-tests. **(A-D)** Average tangential force and EMG data across two pedal cycles (0–720°) at different workloads: (i) for all subjects (subjects) (n = 14) **(A, B)**, (ii) for all subjects vs subject 9 **(C)** and (iii) for all subjects vs group A/B (both n = 7) **(D)**. The RF force/EMG curve differs from VL, TA and ST and reaches comparable activity only at high workloads **(B)**. Simultaneously, the negative tangential force (Fneg) decreases with increasing workload **(A)** (see also Fig 1A). EMG activity of all subjects in the stages before the MLSSw is clearly higher than in subjects 9 **(C)**. Between 39% max and 78% max, RF activity of subject 9 steeply increases in parallel with the average activity of all muscles. In contrast, RF activity across all subjects lags behind the mean EMG of all muscles up to high workload levels **(C)**. RF activity increases clearly at the MLSSw in group A, which is not the case in group B and the overall sample **(D)**. The other three recorded muscles (EMG m3) also tend to be more active in group A than in group B and the overall sample **(D)**. For detailed description of data analysis and parameter definitions see Methods and S1 Fig. Shown is mean.

Intra- and inter-individual comparison of EMG and crank force maxima revealed different, nonlinear curves for lower limb muscles (Fig 7). Interestingly, RF behavior differed from other muscles in terms of EMG/force curve characteristics (Fig 7). While on average, excitation of the VL, ST and TA increased linearly with Ft, the RF became activated only at higher workloads (Fig 7). As already shown for CP-tests (Fig 1A), Fneg decreased with increasing Ft and contribution of the RF (Fig 7A and S7 Fig). The force level at which the RF started to contribute substantially to total force/power output varied between individuals and appeared to depend on cycling experience (Fig 7D). In group A, RF activity increased more steeply in the MLSSw range (S8 Fig). This specific temporal recruitment pattern also applied to activity levels of the VL, ST and TA (Fig 7D and S7 Fig). Importantly, the steep and strong RF activation in CP-tests (Figs 3, 4 and 6), which resulted in reduced negative force (Fneg) and increased total propulsive force (Ft r+l), also occurred during MP-tests (S7 and S8 Figs). However, in MP-tests, this phenomenon only occurred from MLSSw up to the maximum workload (S7 and S8 Figs). This choreography of quadriceps muscle activation in more powerful athletes, both in CP-tests and MP-tests, suggests a strong influence of neural circuits that govern motor pattern generation in highly trained cyclists.

### Distinct recruitment patterns in CP- and ILT-tests

**Fig 8:**
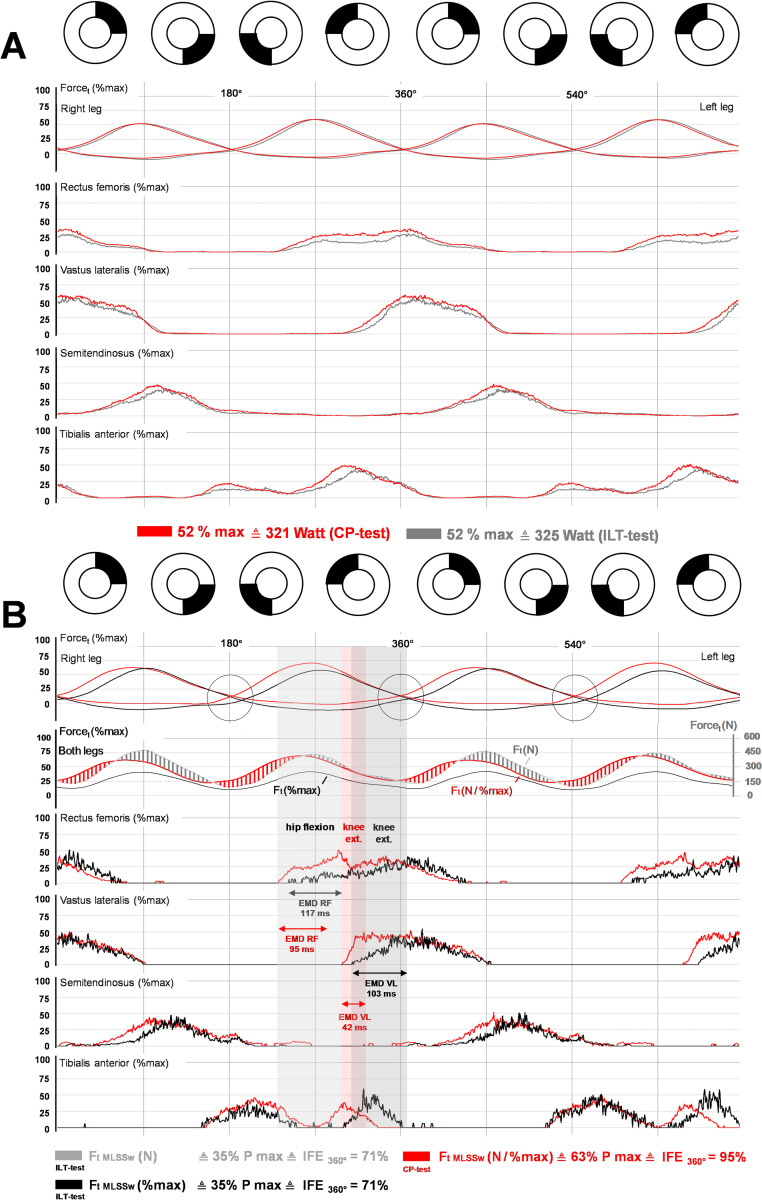
Comparison of CP- and ILT-tests. **(A)** Average normalized bi-pedal crank force and EMG data across two pedal cycles (0–720°) at the MLSSw for ILT-tests (black) vs CP-tests (red) (n = 11). Considering the methodological problems of comparing EMG data recorded on different days, activation of the four recorded lower limb muscles appeared to be earlier and more intense in CP-test. **(B)** Average normalized bi-pedal crank force, total average tangential force and average EMG data for test subject 9 across two pedal cycles (0–720°) at the MLSSw during ILT- (black) and CP-tests (red) **(B, top row)**. **(B, second line)** depicts curves of absolute propulsive force (Newton). Due to the early and strong RF/VL activity during the CP test, the force drops less at TDC and rises more steeply in Q1 than in the ILT-test. Note the different electromechanical delays (EMDs) for RF and VL (for further explanations see Fig 6).

**Fig 9:**
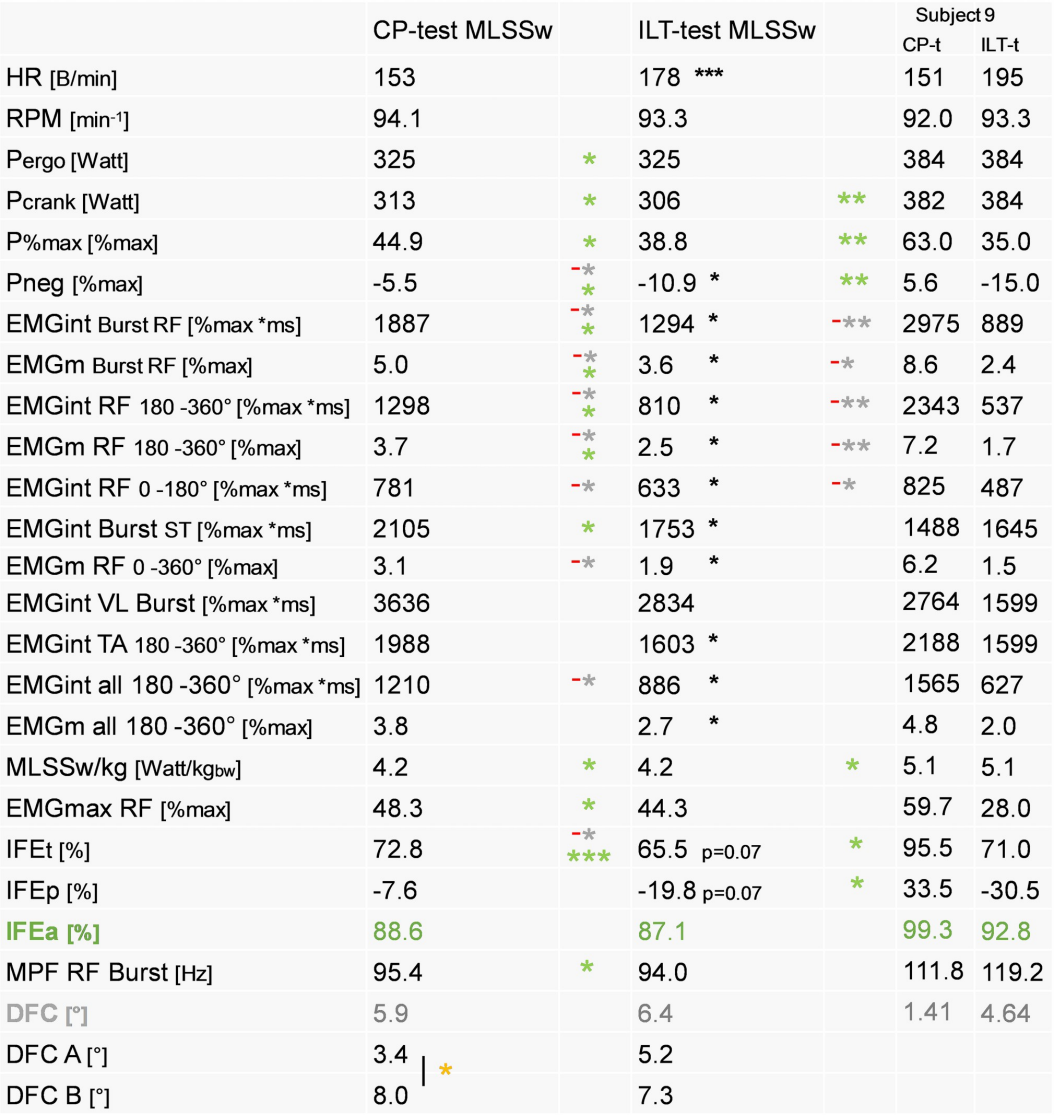
Comparison of parameters relevant for coordination and effective force development in CP- and ILT-tests. Time-based results for ILT- vs CP-tests reveal differences for RF and partially for ST, and TA activity as well as for the average EMG of all 4 recorded muscles (EMGm all) in the second part of the pedal cycle for all examined subjects (n = 11) and for subject 9. The delay of force crossings (DFCs) is inversely correlated to activation behavior of the RF which parallels results from CP-tests (see also Fig 7). Note that the correlation between IFEa and RF-activity between 180°-360° is missing in the ILT-test. P%max and Pneg are shown as mean of both cranks. IFE(t,a,p) is shown as mean of the right crank. For detailed description of data analysis and parameter definitions see Methods and S1 Fig. Grey asterisks (*): correlation to DFC, green asterisks (*): correlation to IFEa, black asterisks (*): two-tailed paired t-tests, yellow asterisks (*): two-tailed unpaired t-tests. Shown is mean. *P < 0.05, **P < 0.01, ***P < 0.001.

Next, we compared CP- and ILT-test data to investigate whether motor patterns in cycling are variable or follow a fixed recruitment scheme. Relative tangential power/force at the MLSS (P%max) differed clearly between ILT- and CP-test (Fig 8), which was well illustrated by subject 9’s data (Figs 8B and 9). Normalization of the total propulsive force (Ft r+l) to absolute power at the MLSS (384 W) revealed a prominent force development in Q1 during CP-tests (red shaded areas). In contrast, during ILT-tests, we found a more pronounced force development in Q2 (grey shaded areas) (Fig 8B). In MP-tests performed for data normalization, subject 9 achieved a maximum power of 1097 W, when the MP-test was executed before the ILT-test ("ILT recruitment pattern"). However, when the MP-test preceded the CP-test ("CP recruitment pattern") the maximum power was 610 W. Consequently, the relative tangential force at the MLSS (P%max) was 35% during the ILT- and 63% during the CP-test. Calculation of IFEs (IFEt = 71% vs 95%), revealed that the resulting/total force (Frs r+l) was approx. 124 N higher under ILT conditions (Frs r+l ILT- / CP-test = 492 N vs 368 N). When considering associated activation parameters, the differences in RF, ST, TA, and EMGm activity in Q3/Q4 were particularly striking (Fig 9).

Compared to CP-tests, activation of RF and VL was delayed and less intense in ILT-tests (Figs 8, 9, S9 Fig). Interestingly, we found such a test-dependent variation of muscle activation predominantly in group A, indicated by the delay of DFCs (Fig 9). This specific activity of lower limb muscles is well illustrated by in-depth analysis of subject 9 (Fig 8B). Due to the comparatively later and less intensive activation of the RF and, partly, of the VL in the ILT-test, more negative force (Fneg) was generated and IFE(t,a,p) decreased (Fig 9). Thus, the correlation between RF EMG parameters in Q3/Q4 and IFE in the CP-test does not exist in ILT-tests (Fig 9). Fig 9 shows the clear negative relationship between DFC and EMG parameters of the RF, especially in the posterior part of the pedal cycle. DFC decreased with increasing and earlier RF/VL activity or correspondingly increased with decreasing and later activity of both muscles.

**Fig 10:**
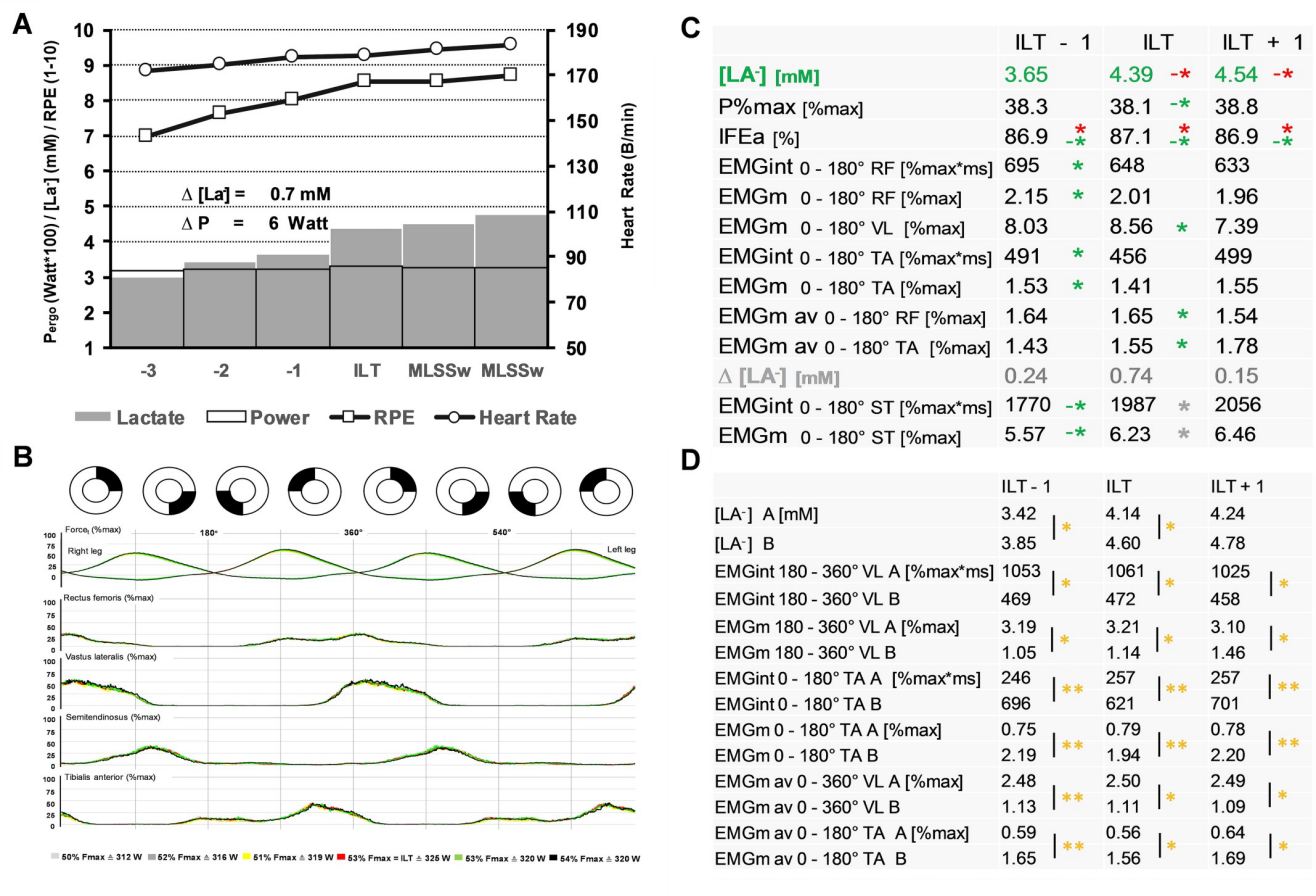
Force, EMG and lactate kinetics around the lactate threshold in ILT-tests. **(A)** Summary of ILT-tests (n = 11). Blood lactate concentration (mM), Pergo (Watt), rate of perceived exertion (RPE, 1-10) and heart rate (beats/min) for the final 6 increments of ILT-tests. A step-like blood lactate accumulation occurs at the individual lactate threshold (ILT, Δ[La^−^] = 0.7 mM) after a slight workload increment (ΔP = 6 Watt). Immediately after a comparable slight workload reduction (ΔP = 5 Watt), lactate accumulation slows down, indicating the maximal lactate steady state workload (MLSSw) [24]. **(B)** Averaged bi-pedal crank force and EMG data across two pedal cycles (0–720°) (n = 11) during three increments before and during two increments after the ILT. There are no visible differences in force and recruitment patterns. **(C, D)** Relationship of capillary lactate concentration [La^−^] to MLSSw/kg, mechanical and EMG parameters **(C)** and differences in lower limb muscle activity between group A and B **(D)** for the pre-ILT stage, at the ILT and for the post-ILT stage, calculated for each revolution. P%max is shown as mean of both cranks. IFEa is shown as mean of the right crank. For detailed description of data analysis and parameter definitions see Methods and S1 Fig. Red asterisk (*): correlation to MLSSw/kg **(C)**, green asterisks (*): correlation to [La^−^] at ILT **(C)**, grey asterisks (*): correlation to Δ[La^−^] **(C)**, yellow asterisks (*): two-tailed unpaired t-tests **(D)**. Shown is mean. *P < 0.05, **P < 0.01.

This altered muscle activation during ILT-tests, which was predominantly observed in group A, also explains the lack of group differences for RF parameters illustrated in Fig 10D. Here, group A and B were only distinguished by the intensity of VL activation and partly by TA activity (compare Fig 1B and 1C). This phenomenon was likely due to the low workload at the beginning of the ILT-test. Presumably, crank force (Ft) was insufficient for strong RF activation during the initial phases of the ILT-test, and possibly this intra- and intermuscular recruitment scheme was maintained throughout the remainder of the test.

The relationship between capillary lactate concentration, force and EMG parameters at the MLSSw is shown in Fig 10C. Here, lactate production was mainly related to RF-, VL-, and TA- activity within the anterior half of the pedal cycle (Fig 10C). However, ST activity at pre-ILT stage was correlated negatively with [La^-^] and positively with Δ[La^-^] at ILT (Fig 10C). Overall, group A athletes had lower lactate levels (Fig 10D). Moreover, we found a negative correlation of MLSSw/kg and IFEa with [La^**−**^], while IFEa was positive correlated to MLSSw/kg (Fig 10C). General recruitment patterns were equal around the ILT (Fig 10A). However, in some cases, specific recruitment patterns at the ILT were observed, for example a sudden increase of TA activation in subject 9 (S10 Fig).

## Discussion

The transition from moderate to heavy exercise is characterized by a rapid onset of physical exhaustion. The exact determination of this individual threshold intensity is crucial for setting optimal training stimuli and choosing the highest possible load in competition that does not lead to premature exhaustion [23, 24, 35]. Physiological parameters used in "threshold determination" such as respiratory gas exchange values or lactic acid concentration [36–38] are cellular end products of a more or less specific neuronal composition process of motor units grouped in muscles with the aim of generating force. Simply, they are the result of "muscle coordination" or “motor control” (for more details see [24, 35]). The reciprocal interplay between intra-/inter-muscular coordination and energy metabolism determines both the task-specific optimization of movement, and the cellular adaptation processes that depend on it [39]. Thus, knowledge of superior performance-related and controllable recruitment patterns can help athletes learn and optimize their sport. Cycling is particularly well suited for coordination studies due to its relatively simple and fixed movement pattern. To probe muscle coordination and crank force in trained and highly trained cyclists, we recorded the activity of 4 representative leg muscles (RF, VL, ST, and TA) and tangential/radial crank forces at both cranks under three test conditions: (i) maximal power (MP-tests, (ii) ILT-tests, and (iii) three 4-min constant power (CP)-tests at ± 10% MLSSw (for detailed description see “Methods” section).

Consistent with previous data [14–16], CP-tests revealed considerable inter-individual redundancy in recruitment behavior of the four recorded muscles, particularly of the RF, with apparently uniform force progression at both cranks (S5 Fig). However, more detailed analyses demonstrated clear relationships between quadriceps muscle (RF/VL) coordination patterns and individual endurance levels (MLSSw/MLSSw/kg) (Fig 1B), as well as for performance-relevant force parameters at the crank (Figs 5 and 9). Basically, cyclists of group A innervated their RF more strongly and earlier during hip flexion in the fourth quadrant (Q4) of the pedal cycle (Figs 2–6, S6 Fig). The VL was also used earlier and together with the RF during knee extension (Figs 2, 3 and 6). Obviously, this activation strategy can offer an advantage, at least when the highest possible power has to be applied over a longer period of time (e.g. at the MLSSw [24]), which is the case in 1-h track racing [25] or time trial races [26, 27].

Considering force parameters related to individual endurance performance (MLSSw/MLSSw/kg) and innervation parameters, we found that three variables were of major interest: (i) negative force/power (Fneg/Pneg), (ii) IFE, and (iii) delay of force crossings (DFC) (Figs 2–5 and 9). Fneg was a part of IFE calculation (see S1 Fig) and, thus, both parameters had a similar relationship to innervation parameters (Figs 5 and 9). DFC indicated the force development at both pedals at the TDC, and was inversely related to the total propulsive force (Ft r+l). None or low DFC, resulted in more constant propulsion (Figs 2–4, 6B and 8B). At the same time, the negative force decreased in Q3/Q4 while the IFE increased in the same range (IFEa), and over an entire pedal cycle (IFEt) (Figs 5 and 9). Finally, these changes in crank force were closely related to the timing and intensity of RF activation (Figs 2–4 and 6B). Consequently, RF recruitment pattern determined the direction of force acting on the pedals and, thus, IFE. How did the RF do this?

Two-jointed muscles like the RF are generally assumed to have the ability to determine the direction of joint forces and to do so from proximal to distal and vice versa [40]. The RF is assumed to be a major contributor to overall propulsion around the TDC [12, 16, 41, 42]. However, the temporal decoupling between RF activity in Q3/Q4 and the clearly delayed maximal force development in Q1/Q2 reported in a previous study is difficult to explain [43], but can likely be addressed by simultaneous force recordings at both cranks [16]. Our data show that the crossing points of right and left tangential forces (Ft r/l) could only be determined by bi-pedal/crank force measurements, thus allowing the observation of crucial coordination patterns.

The rectus femoris (RF) supports hip flexion together with the psoas major, iliacus, and vastus intermedius (VI) [40, 44–46] and knee extension together with the VI, vastus medialis (VM), and vastus lateralis (VL) [40, 45]. Individual excitation patterns of the RF were previously described as highly variable [15, 16, 47]. We recorded single and double bursts of the RF ranging from 212° to 109° (Fig 4, S5 and S9 Figs) and found excitation bursts supporting both hip flexion and knee extension (Fig 4, S5 and S9 Figs), or only one of the two movements (S5 Fig). The previous observation that hip flexion at an average workload of 40-55% VO2max with normal pedals resulted in lower RF activity than with clipless pedals [40], emphasizes the direct influence of RF activity on crank force in the second half of the pedal cycle. Our data reveal a positive correlation between RF activity and negative force in the posterior half of the pedal cycle (Figs 1D, 5, S7 and S8 Figs), highlighting the function of the RF as a strut between hip flexors (iliacus/psoas) and the lower leg. The RF and VI [45] can unload the rear pedal and generate a level of tension in Q3/Q4, that is sufficient to produce positive tangential forces in the rear, although a powerful downstroke occurs diagonally in front (Figs 1A, 2–4, 6 and 8B). Subsequently, knee extension occurs through joint activation of all four quadriceps heads [45]. Consistent with an EMG study of untrained cyclists in which RF activity derived from intramuscular recordings [48], we found that some athletes innervated their RF continuously within a long burst from the beginning of hip flexion until the end of knee extension (Fig 4 and S5 Fig), while other athletes produced a double burst, separated by a silent period (Fig 4 and S9 Fig). Both recruitment strategies generated RF tension during hip flexion, which shortened the delay between innervation and transmission of force (electromechanical delay, EMD). If the switch from pulling to stretching of the RF was interrupted by a silent period (double bursts, Fig 4 and S9 Fig), we found EMDs for RF and VL that were similar to the single burst pattern. In this case, force development was only delayed by the silent period (Fig 4 and S9 Fig). We also observed that minor or absent pre-activation of the RF, further delayed force development during leg extension. Such a delayed force development manifested itself by a high DFC and caused a transient drop in total propulsion (Fig 6B). Group A athletes who unloaded the rear pedal in whatever way using the RF, were characterized by force transmission through the RF/VL and probably all quadriceps muscles at earlier stages during the subsequent knee extension (Figs 2–4 and 6). Group B athletes who had minor RF activation in Q3/4, produced more negative force in this section (Figs 1A, 5, S6). In the latter case, the rear leg was pushed upwards by its counterpart and the force development within the knee extension occurred later (Figs 4 and 6B). Subject 9’s RF coordination (Fig 6A) appeared to be particularly sophisticated. The subject preloaded its RF in Q3/Q4 through strong activation (Fig 6A) and was able to effectively transfer this stored energy together with the other quadriceps muscles into knee extension (Fig 6A). This observation was supported by the minimal EMD (42 ms) between the RF/VL EMG and the onset of force development in the quadriceps, as well as the considerable force production in Q1 (Fig 6A and S6 Fig).

Considering the RF recruitment pattern found here, we conclude that the RF is not critical for generating additional propulsive forces by contributing pulling up the rear pedal. Rather, the RF should be brought in an optimal preload mode, allowing it to rapidly push the crank over the TDC with minimum force and energy loss, and then initiate early knee extension in concert with all quadriceps muscles.

We found that subject 9’s (time trial specialist on Tour de France level) muscle recruitment pattern clearly differed from the other subjects not only in RF coordination, but also in a short-lasting co-contraction between VL and ST (S5 Fig), and in a balanced EMG intensity (approx. 50% max) in all four recorded muscles (Figs 3, 4 and 8B, S5 Fig). Additionally, we observed that subject 9 achieved the highest mechanical efficiency (IFEt = 95%) in CP-tests (Fig 9).

The data shown above suggest two patterns of RF activation: variable but pronounced RF activation and minor or no RF activation. Strong RF activation unloads the rear pedal and initiates leg extension with support of all quadriceps muscles more rapidly and effectively. The former pattern is more common in highly trained athletes and is particularly pronounced in a top international cyclist. Such an advantageous pedaling technique is probably acquired through training, and if this hypothesis is true, this coordination pattern should also be found in MP-tests. The analysis of the force development of all subjects during MP-tests (Fig 7A and 7B) revealed a non-linear recruitment pattern of all recorded muscles, in particular of the RF. Compared to the other muscles, we found that the RF was significantly less active at low workloads and only joined the other muscles at workloads above 90% Ftmax (Fig 7B). Obviously, the basic motor program for cycling involves the RF in force production only at higher workloads. Interestingly, such a sigmoidal progression of EMG/force relationship has also been described during isometric knee and leg extension movements [49].

Group comparison of RF activity in the MLSSw range revealed a significantly larger EMG/force quotient in highly trained athletes (Fig 7D). This phenomenon was most striking in subject 9 (Fig 7C). Here, we observed that the excitation intensity of all recorded muscles, but especially of the RF, increased dramatically between 39% and 78% Ftmax. However, further force progression up to 100% Ftmax was much less pronounced in all muscles (Fig 7C). At linearly increasing power in the MLSSw range, the data revealed similar recruitment patterns in CP- and MP-tests (see also S7 and S8 Figs). This suggests that bi-pedal coordination at the MLSSw, and RF activation in particular, have significant impact on cycling performance.

We sometimes found different coordination patterns in ILT- and CP-tests (Figs 8 and 9, S9 Fig), although the test setup (cadence, body position, electrode position, workload) was identical. Kautz and colleagues studied 14 elite cyclists during the transition from moderate (61% VO2max) to heavy exercise (92% VO2max) and revealed that the workload increment was accompanied by a change in pedaling technique and decreased negative force [50]. Surprisingly, there were two different “new” pedaling techniques at higher workloads. In seven athletes, Kautz et al. [50] registered increased vertical force in the first half of the pedal cycle. For the other half of participants, they recorded increased horizontal force in Q1 [50]. Our study also found that at high workloads (MLSSw), some group A athletes used a different pedaling technique in the ILT-test than in CP-tests (Figs 8 and 9, S9 Fig). The MLSSw is widely accepted as a precise and valid reference point to set training stimuli and to adjust the optimal workload during competition [24, 51]. However, our data suggest non-fixed coordination patterns at higher workloads, which in turn can have a major impact on metabolism and neuromuscular adaptation mechanisms.

Our findings of varying coordination strategies in high-intensity cycling may have their origin in the different test procedures. CP-tests were characterized by instantly high workloads set to MLSSw ± 10%. In contrast, the ILT- test protocol did not allow for anticipation of the workload or even the individual threshold range. Here, subjects were guided into the MLSSw range via several steps with increasing workload [24]. We thus hypothesize that the test differences reflected a generally more cautious, wait-and-see mental attitude during ILT-tests, which may influence sensorimotor feedback loops and/or the selection of the appropriate coordination program if it has been previously learned. The ILT-test usually starts at a workload that causes low RF activation, so that central muscle control is predetermined and possibly stable throughout the test, making adjustments of motor control difficult. In line with this assumption, it has been shown for isometric isotonic VL contractions that the CNS does not change the recruitment pattern at a constant intensity of 20%max until exhaustion [52]. Two recent studies support our findings that more powerful cyclists generate less negative force (Fneg) at comparable workloads [22] and that hip flexors are involved in such a pedaling technique [5, 22]. Garcia-Lopez et al. [22] found less negative force and larger hip flexion angles in professional cyclists compared to amateurs. Similarly, Leary et al. [5] demonstrated that cyclists with a high lactate threshold (LT) used their hip muscles significantly more during high-intensity cycling (80% to 90% VO2max) than athletes with a lower LT. Moreover, comparable to our study, athletes with a high LT had lower capillary lactate concentrations ([5], Fig 10D). All this suggests that shifting muscle activity from distal to proximal improves movement economy. Apparently, highly trained athletes can innervate their large hip flexors and extensors stronger and earlier in high-intensity cycling, resulting in reduced force load per muscle cross-section [5] and in adequate stimuli for the development of a muscle fiber structure optimized for endurance loads. These assumptions are supported by the findings that highly endurance-trained cyclists have a higher FT I fiber content in the VL [3, 9] and that the six hip flexors and knee extensors (iliacus, psoas, RF, VI, VL, and VM) account for approximately 33% of the total lower limb muscle mass (35 major muscles) [53].

Taken together, previous data and our results suggest the existence of a pedaling technique that can be considered economical for endurance exercise and one that is more advantageous for short duration peak exercise such as sprinting. The "endurance" technique seems to be a mixture of a specific quadriceps femoris activation (long activation times at low Ft%max, short EMDs, low or minor DFCs), high FT I fiber content in relevant muscles and a major contribution of proximal large muscles. In contrast, we and others suggest a coordination pattern in which the extension force starts later and the contribution of gravitational and inertial forces within the extension movement to overall propulsion increases [54, 55]. Such a delayed extension movement probably incorporates the body weight as a non-muscular component more extensively resulting in higher crank forces (see also Figs 3, 4, 6B and 8B). These are necessary for short-term maximum loads such as sprints, but cannot be maintained over longer periods of time due to muscular fatigue. Consistent with these findings, elite sprinters were reported to have a significantly higher maximal power output, a higher VL volume and a larger VL pennation angle than elite endurance athletes [56].

The assumption that low RF activation during high-intensity cycling is more fatiguing and associated with higher tangential and radial crank forces is supported by a recent study with untrained participants who performed a 45-minute cycling test at a constant workload of 75% VO2max [12]. Theurel and colleagues found that participants who received force feedback and were instructed to pull during the upstroke phase of the pedal cycle had higher power maxima at the end of the test [12]. Accordingly, we found that a pedaling technique which was characterized by low RF activation was associated with higher negative force and, consequently, a lower mechanical effectiveness (IFEt) (Figs 8B and 9).

Our recordings revealed that a higher ST activity, was inversely related to MLSSw/MLSSw/kg (Fig 1B) and had apparently no performance enhancing effects, at least in the MLSSw range. This may reflect increased co-contraction of the hamstrings together with the quadriceps (knee extensors) and gluteus maximus (hip extensors) [57, 58], which was necessary to bring the pedal beyond the bottom dead center (S5 Fig).

When pronounced RF activity occurred during the upstroke at the end of the ILT-test (MLSSw range), capillary lactate concentrations were positively correlated to RF, VL and TA activity in Q1/2 and negatively correlated to ST activity in Q1/2 (Fig 10C). In context with the inverse correlation of ST activity with MLSSw/MLSSw/kg, this suggests signs of fatigue in the RF, VL and TA muscles, documented by the capillary lactate concentrations. In addition, we observed a positive relationship between Δ[La^-^] and ST activity (Fig 10C), suggesting that higher ST activity with concomitant fatigue of RF, VL, and TA, causes a more rapid lactate accumulation during the transition from moderate to heavy exercise (MLSSw). Subject 9 used a different pedaling technique in the ILT-test than in the CP-test. In the ILT-test, RF activity was lower in Q4 and force production therefore shifted to Q2 (Figs 8B and 9). At the LT, this also explains that he tried to bring the pedal over the TDC with increased use of the TA and only uses the RF for this purpose after reducing the workload by 7 watts (S10 Fig).

In summary, the coordination pattern characterized by low RF activity in Q3/Q4 appeared to be more fatiguing than the "time trial" pattern found in group A and in particular in subject 9. Accordingly, the latter pedaling technique may also improve movement economy. However, previous studies do not confirm this hypothesis [12, 13].

GE, defined as the ratio between mechanical output and energy expenditure [9] is often used to capture the economy of cycling [6, 59]. Although there are several lines of evidence demonstrating that world-class cyclists exhibit an exceptionally high GE [6–8], which is linked to a high FT I fiber content in thigh muscles [3, 9], short-term variation of pedaling technique (e.g. deliberately pulling up the pedal) with [12] or without [13] feedback, however, does not improve GE. This lack of correlation between IFE and GE in new pedaling patterns is probably due to delayed adaptation of neuromuscular structures/metabolism to acutely changing demands, which require specific training over longer periods of time [59–61]. Furthermore, "pulling up" the pedal even under force feedback conditions cannot automatically be assumed to be a functionally effective innervation. In pilot experiments (unpublished) we observed that negative force in the posterior half of the pedal cycle can also be minimized by the hamstrings (e.g. semitendinosus). Therefore, oxygen consumption and lactate formation may be reduced when adaptation processes in response to homeostatic perturbations (e. g. new and technically effective pedaling technique) have been completed. Consequently, adaptation of GE in response to consciously altered recruitment patterns may be achieved in the course of a prolonged, intensive, and highly specific training process.

In conclusion, we provide evidence for a relationship between individual endurance performance and bilateral leg coordination during cycling, mediated primarily by orchestrated but finely tuned activation of the RF and other hip flexors and knee extensors. We hypothesize that highly endurance trained athletes preload their bi-articular RF in the posterior half of the pedal cycle to avoid a drop in propulsion at the TDC. This recruitment pattern reduces negative force and primes an earlier and longer activation of the large hip and thigh muscles (here RF and VL) and additionally increases the likelihood of generating physiological stimuli for neuromuscular adaptation to higher endurance capacity [60]. Comparable to constant workloads, RF activation is also more pronounced during continuously increasing workloads (MP-tests) in highly endurance trained athletes, which may result from intensive training in the threshold range or conscious manipulation of muscle coordination. Knowledge of performance-relevant coordination patterns suggests the use of force/EMG feedback systems to promote motor learning in sports and rehabilitation.

## Supporting information

S1 Fig Parameter definitions [41, 62, 63]. (TIF)

S2 Fig Summary of ILT-tests. Blood lactate concentration (mM), Power (Watt), rate of perceived exertion (RPE, 1-10) and heart rate (beats/min) for the last 6 increments of ILT-tests (n=14). A step-like blood lactate accumulation occurs at the individual lactate threshold (ILT, Δ[La−] = 0.8 mM) after a slight workload increment (ΔP = 6 Watt). Immediately after a comparable slight workload reduction (ΔP = 5 Watt), lactate accumulation slows down, indicating the maximal lactate steady state workload (MLSSw) [24].
(TIF)

S3 Fig Experimental setup. **(A)** Electrode positions and fixation method for rectus femoris, semitendinosus, tibialis anterior and vastus lateralis. **(B)** PowerTec® system for two-dimensional force measurement, mounted on a bicycle crank arm. **(C)** Bipolar EMG electrode (left) and reference electrode (right). **(D)** EMG–Ag/AgCl electrode with a special blunted adhesive strip. **(E)** E3 Optical Encoder Kit (US Digital ®) integrated into the crank axis.
(TIF)

S4 Fig Experimental setup and data acquisition. (TIF)

S5 Fig Examples for redundancy and diversity of recruitment patterns in CP-tests. Averaged bi-pedal crank force and EMG data across two pedal cycles (0–720°) at MLSSw -10% (yellow), MLSSw (red), MLSSw +10% (green (for subject 9 **(A)**, subject 10 **(B)**, subject 12 **(C)** and subject 6 **(D)**. There are large interindividual differences with respect to (i) the timing of the RF as hip flexor (dark gray shaded areas) and the timing as knee extensor (light gray shaded areas), (ii) with respect to the co-contraction periods between RF and VL during knee extension, and (iii) with respect to the co-contraction periods between the knee extensors RF/VL and the knee flexor ST (green shaded areas). **(E)** Comparison of innervation times for group A vs group B. Note the high interindividual variability in activation duration (ID) as well as for start (IB) and termination (IE) of muscle activation. Yellow asterisks (*): two-tailed unpaired t-tests (E) For detailed description of data analysis and parameter definitions see Methods and S1 Fig.
(TIF)

S6 Fig Recruitment patterns expressed as means and for individual subjects in CP-tests. (**A)** Averaged bi-pedal crank force- and EMG data across two pedal cycles (0–720°) at MLSSw–10% (yellow), MLSSw (red) and MLSSw + 10% (green) (n = 14). Note the clear differences in EMG amplitudes for RF and VL in particular at the MLSSw and MLSSw + 10%. (**B)** Data analysis for segments (Q1-Q4) of the pedal cycle reveals strong correlation for force and RF/VL activity to MLSSw/ MLSSw/kg in Q1/Q4. (**C)** Comparison of data from athletes of different performance categories. Subject 9 (MLSSw/kg = 5.1 W/kgbw) clearly differs from the other athletes in force production and recruitment patterns. Subject 9 develops its highest propulsive force in Q1, without generating negative force in Q3/Q4. Subjects 10/11 (4.1/4.4 W/kgbw) reach their force maxima in Q2 and generate considerable negative force in Q3/Q4. Force distribution in Subject 9 underlies a specific recruitment pattern: (i) high RF and VL activity which is mainly achieved by their early activation in the second half of the pedal cycle, and (ii) comparatively low VL, TA and ST activity in Q1/Q2. For the least powerful Subject 12 (MLSSw = 3.5 W/kgbw) we recorded the highest propulsive force in Q2 with strong activation of the VL in Q1, of the ST in Q2 and of the TA in Q4. For detailed description of data analysis and parameter definitions see Methods and S1 Fig. Black asterisks (*): two-tailed paired t-tests, red asterisks (*): correlation to MLSSw/kg, green asterisks (*): correlation to MLSSw. Shown is mean. *P < 0.05, **P < 0.01, ***P < 0.001.
(TIF)

S7 Fig Force/EMG pattern during MP-tests depends on endurance performance level. **(A-D)** Averaged tangential force and EMG data across two pedal cycles (0-720°) at different workloads (MP-tests) for subject 9 and group A/B (both n = 7). Pedaling technique clearly differs between subjects/groups in the maximum force/workload range **(A, B)**, and at the transition from moderate to heavy domains of exercise **(C, D)**. As with constant power (CP)-tests, we found stronger activation of the RF and VL in highly trained athletes, which reduces negative force (Fneg) in the second half of the pedal cycle **(A)**, in favor of total propulsive force (Ftmax r+l) **(B)**. This obviously advantageous muscle coordination pattern at the MLSSw also becomes apparent by comparison of group A vs group B **(C)** and subject 9 vs group B **(D)**. For detailed description of data analysis and parameter definitions see Methods and S1 Fig.
(TIF)

S8 Fig Force/EMG pattern of lower limb muscles during MP-tests. **(A-E)** Averaged tangential force and EMG data across two pedal cycles (0-720°) for five increments in MP-tests. Group A (green), group B (black) and subject 9 (red). For detailed description of data analysis and parameter definitions see Methods and S1 Fig.
(TIF)

S9 Fig Comparison of CP- and ILT-test of subject 11. Averaged normalized bi-pedal crank force and EMG data across two pedal cycles (0–720°) at the MLSSw for ILT-tests (black) vs CP-tests (red) of subject 11 (top row). Second line depicts curves of absolute propulsive force (Newton). Note the longer delays of force crossings (DFCs), the different electromechanical delays (EMDs) for RF and VL and the shift in force development from Q1 to Q2 in the ILT-test.
(TIF)

S10 Fig Pattern of lower limb muscle activity during an ILT-test at the ILT/MLSSw in a professional cyclist (subject 9). **(A)** Blood lactate concentration (mM), Power (Watt), rate of perceived exertion (RPE, 1-10) and heart rate (beats/min) for the final 6 increments of the individual lactate threshold (ILT)-test. A step-like blood lactate accumulation occurs at the individual lactate threshold (ILT, Δ[La^−^] = 0.7 mM) after a slight workload increment (ΔP = 10 Watt). Immediately after a comparable slight workload reduction (ΔP = 7-10 Watt), lactate accumulation slows down, indicating the maximal lactate steady state workload (MLSSw) [24]. **(B)** Averaged tangential force and EMG data across two pedal cycles (0–720°) for the pre-ILT stage, at the ILT and at the MLSSw. Note the elevated EMG activity of the TA at the ILT. One increment later (MLSSw), TA activity and negative tangential force (Fneg) are reduced, while early RF activation is enhanced. For detailed description of data analysis and parameter definitions see Methods and S1 Fig.
(TIF)
