# Supplementary figures and images for: A quadriceps femoris motor pattern for efficient cycling
Source: PLoS One. 2023 Mar 16;18(3):e0282391. doi: 10.1371/journal.pone.0282391 (PMC10019633; doi:10.1371/journal.pone.0282391)

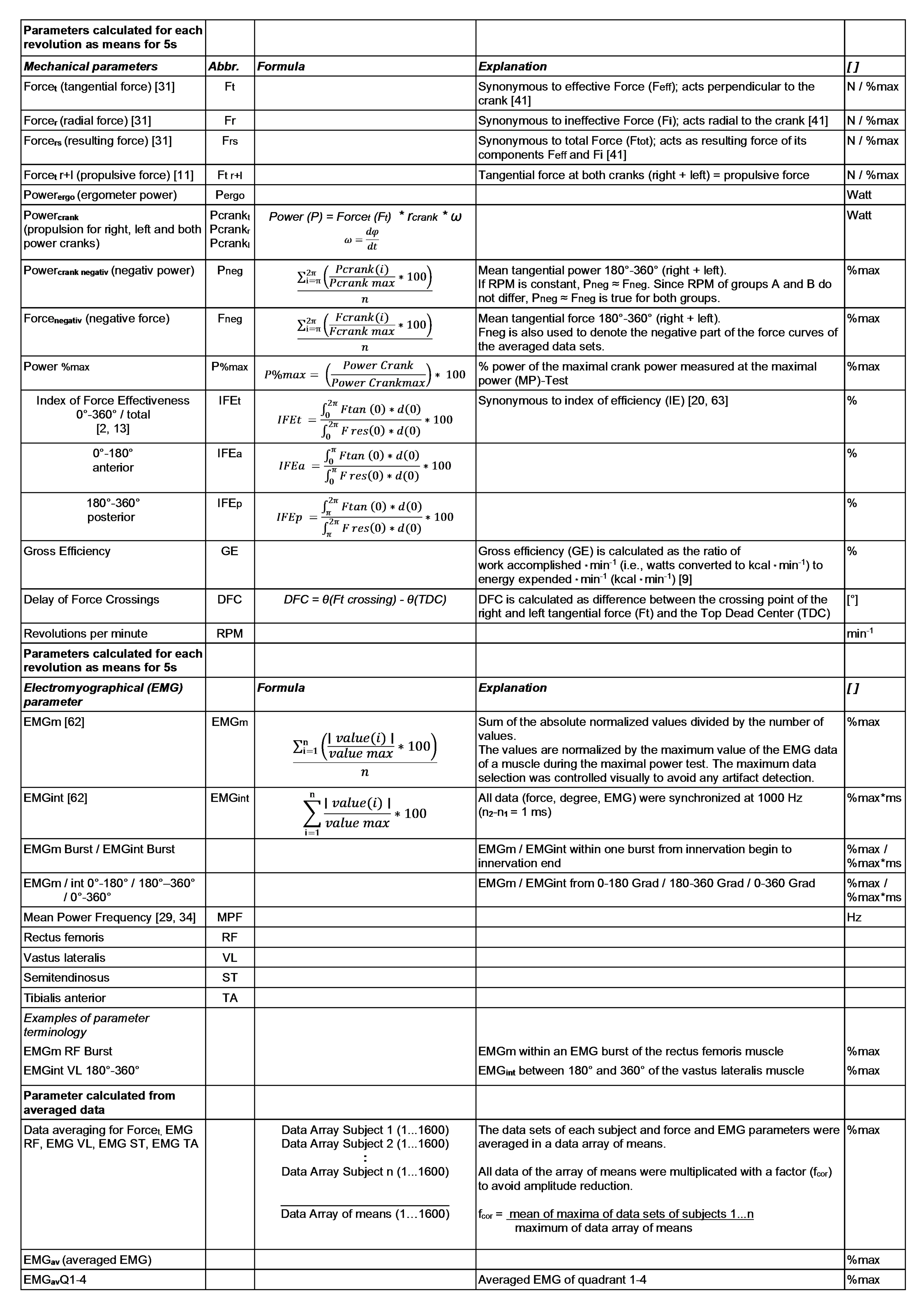

Supplement: S1 Fig — (TIF) [file pone.0282391.s001.tif]

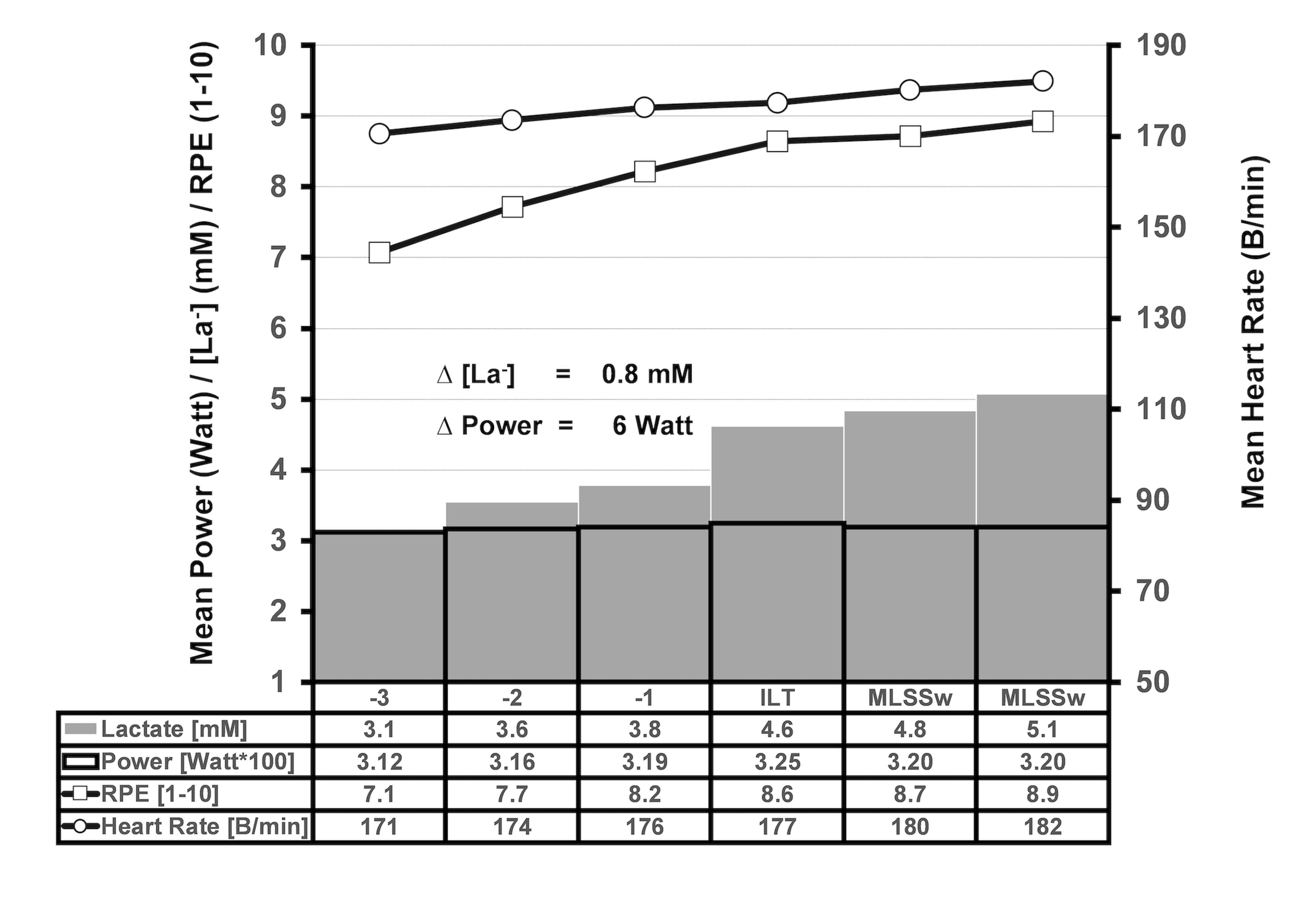

Supplement: S2 Fig — Blood lactate concentration (mM), Power (Watt), rate of perceived exertion (RPE, 1-10) and heart rate (beats/min) for the last 6 increments of ILT-tests (n=14). A step-like blood lactate accumulation occurs at the individual lactate threshold (ILT, Δ[La−] = 0.8 mM) after a slight workload increment (ΔP = 6 Watt). Immediately after a comparable slight workload reduction (ΔP = 5 Watt), lactate accumulation slows down, indicating the maximal lactate steady state workload (MLSSw) [24]. (TIF) [file pone.0282391.s002.tif]

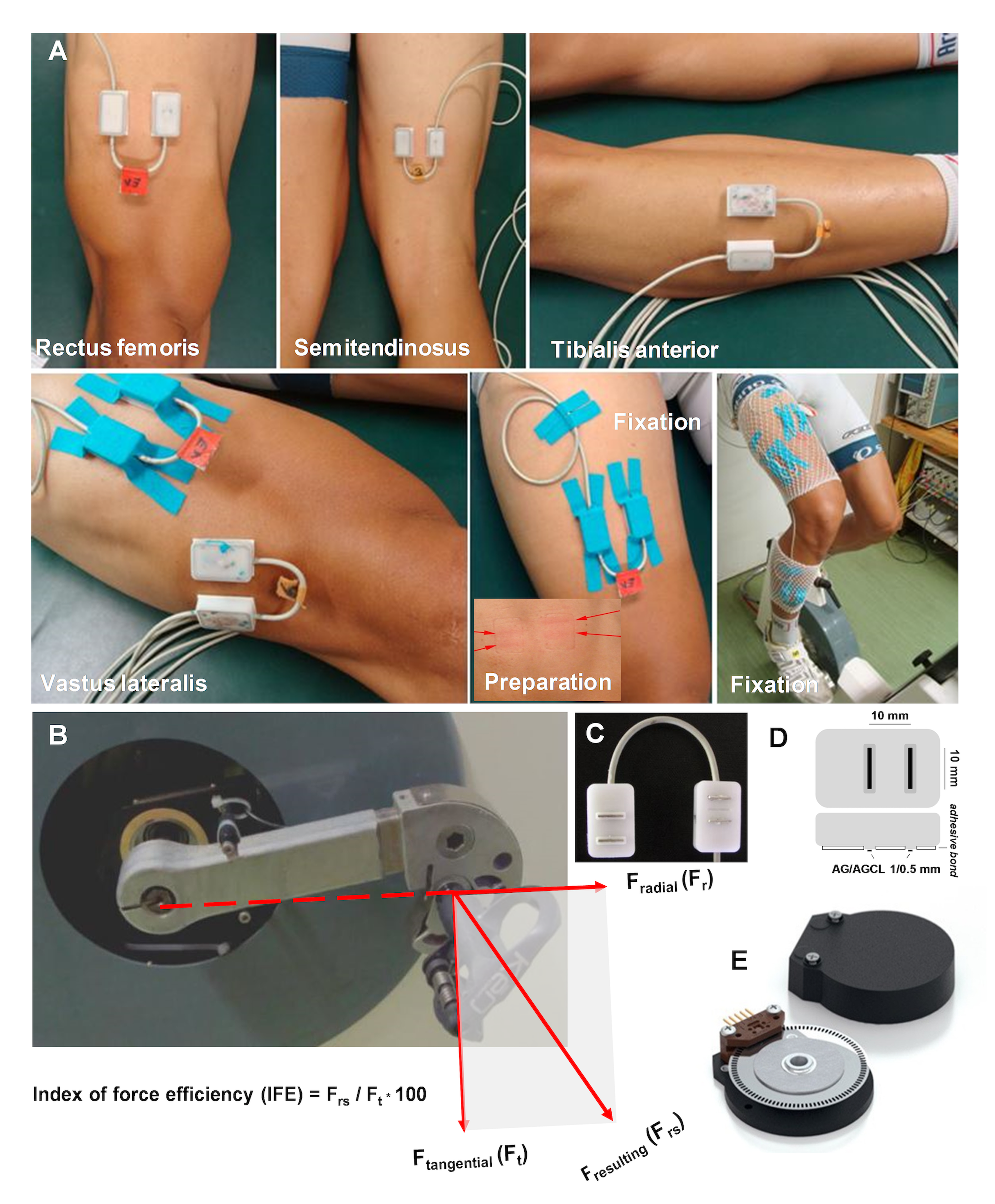

Supplement: S3 Fig — (A) Electrode positions and fixation method for rectus femoris, semitendinosus, tibialis anterior and vastus lateralis. (B) PowerTec® system for two-dimensional force measurement, mounted on a bicycle crank arm. (C) Bipolar EMG electrode (left) and reference electrode (right). (D) EMG–Ag/AgCl electrode with a special blunted adhesive strip. (E) E3 Optical Encoder Kit (US Digital ®) integrated into the crank axis. (TIF) [file pone.0282391.s003.tif]

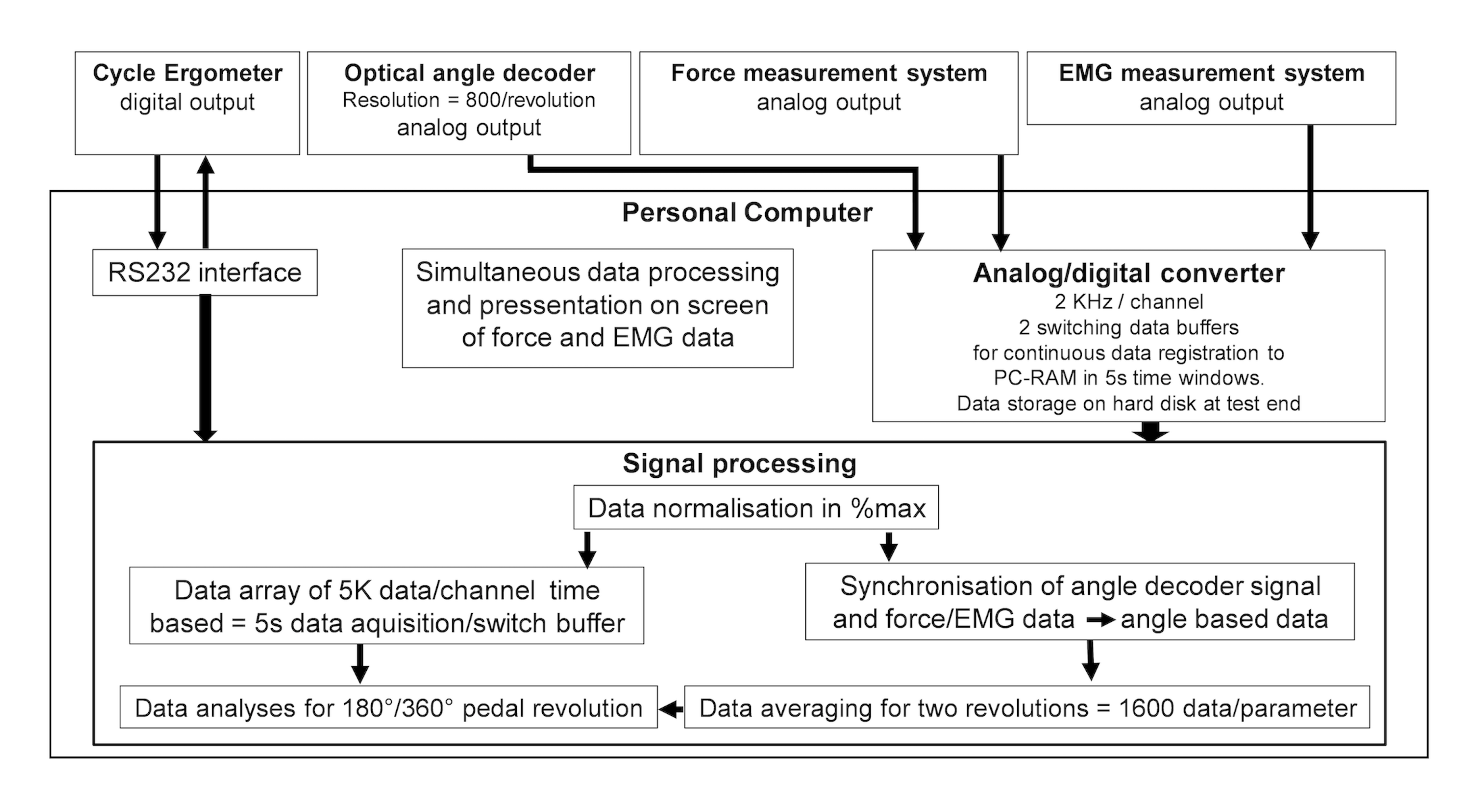

Supplement: S4 Fig — (TIF) [file pone.0282391.s004.tif]

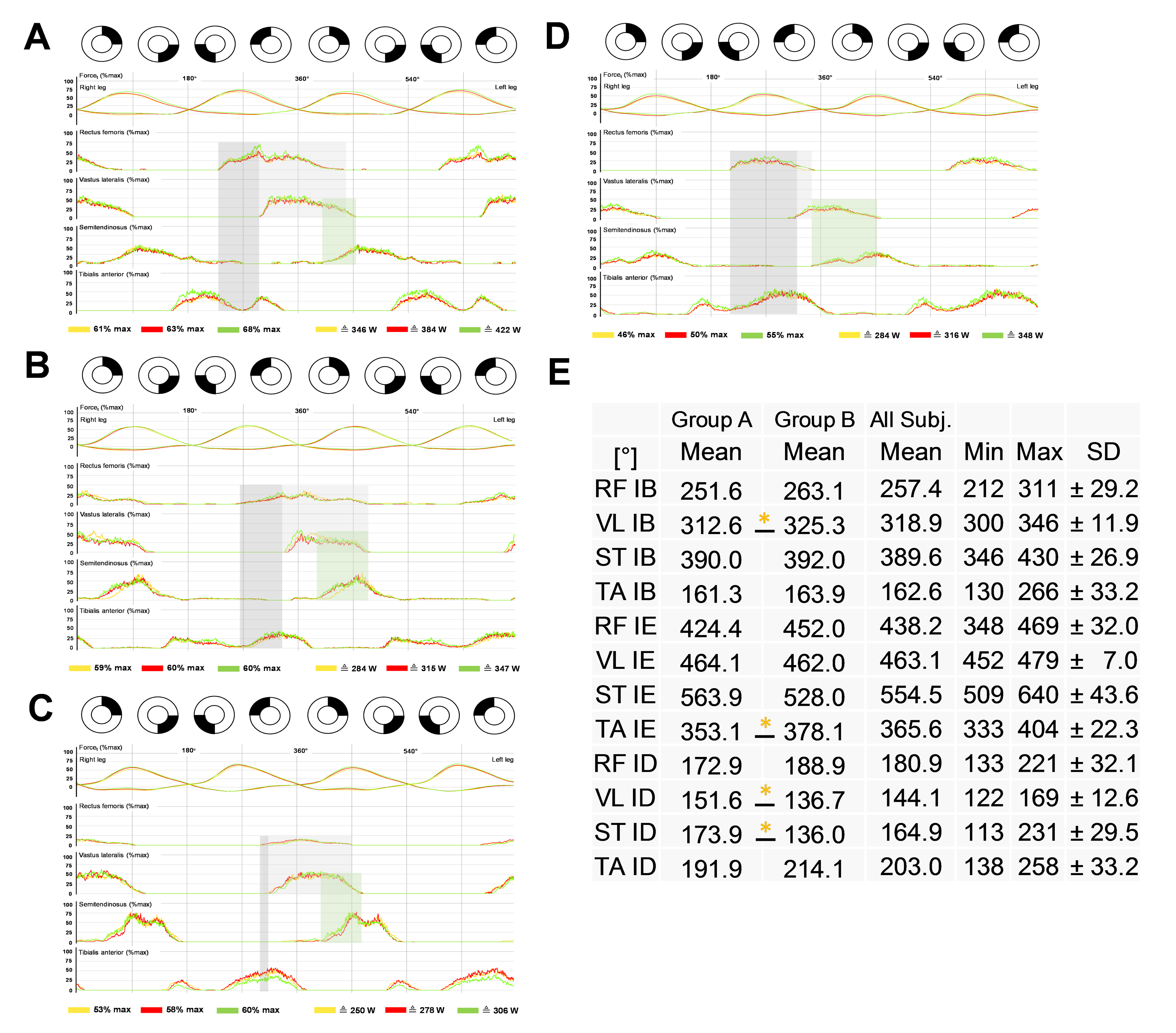

Supplement: S5 Fig — Averaged bi-pedal crank force and EMG data across two pedal cycles (0–720°) at MLSSw -10% (yellow), MLSSw (red), MLSSw +10% (green (for subject 9 (A), subject 10 (B), subject 12 (C) and subject 6 (D). There are large interindividual differences with respect to (i) the timing of the RF as hip flexor (dark gray shaded areas) and the timing as knee extensor (light gray shaded areas), (ii) with respect to the co-contraction periods between RF and VL during knee extension, and (iii) with respect to the co-contraction periods between the knee extensors RF/VL and the knee flexor ST (green shaded areas). (E) Comparison of innervation times for group A vs group B. Note the high interindividual variability in activation duration (ID) as well as for start (IB) and termination (IE) of muscle activation. Yellow asterisks (*): two-tailed unpaired t-tests (E) For detailed description of data analysis and parameter definitions see Methods and S1 Fig. (TIF) [file pone.0282391.s005.tif]

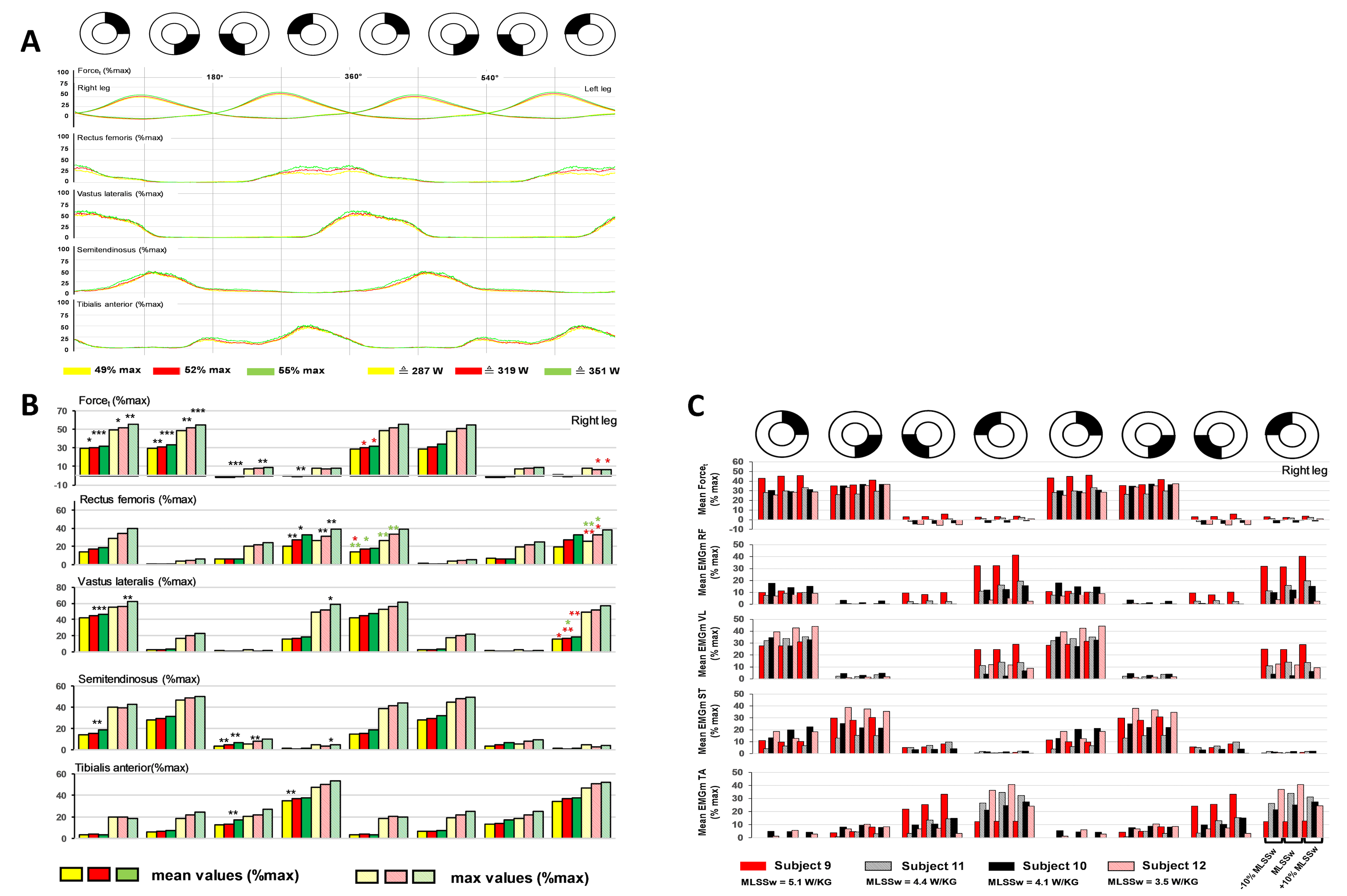

Supplement: S6 Fig — (A) Averaged bi-pedal crank force- and EMG data across two pedal cycles (0–720°) at MLSSw–10% (yellow), MLSSw (red) and MLSSw + 10% (green) (n = 14). Note the clear differences in EMG amplitudes for RF and VL in particular at the MLSSw and MLSSw + 10%. (B) Data analysis for segments (Q1-Q4) of the pedal cycle reveals strong correlation for force and RF/VL activity to MLSSw/ MLSSw/kg in Q1/Q4. (C) Comparison of data from athletes of different performance categories. Subject 9 (MLSSw/kg = 5.1 W/kgbw) clearly differs from the other athletes in force production and recruitment patterns. Subject 9 develops its highest propulsive force in Q1, without generating negative force in Q3/Q4. Subjects 10/11 (4.1/4.4 W/kgbw) reach their force maxima in Q2 and generate considerable negative force in Q3/Q4. Force distribution in Subject 9 underlies a specific recruitment pattern: (i) high RF and VL activity which is mainly achieved by their early activation in the second half of the pedal cycle, and (ii) comparatively low VL, TA and ST activity in Q1/Q2. For the least powerful Subject 12 (MLSSw = 3.5 W/kgbw) we recorded the highest propulsive force in Q2 with strong activation of the VL in Q1, of the ST in Q2 and of the TA in Q4. For detailed description of data analysis and parameter definitions see Methods and S1 Fig. Black asterisks (*): two-tailed paired t-tests, red asterisks (*): correlation to MLSSw/kg, green asterisks (*): correlation to MLSSw. Shown is mean. *P < 0.05, **P < 0.01, ***P < 0.001. (TIF) [file pone.0282391.s006.tif]

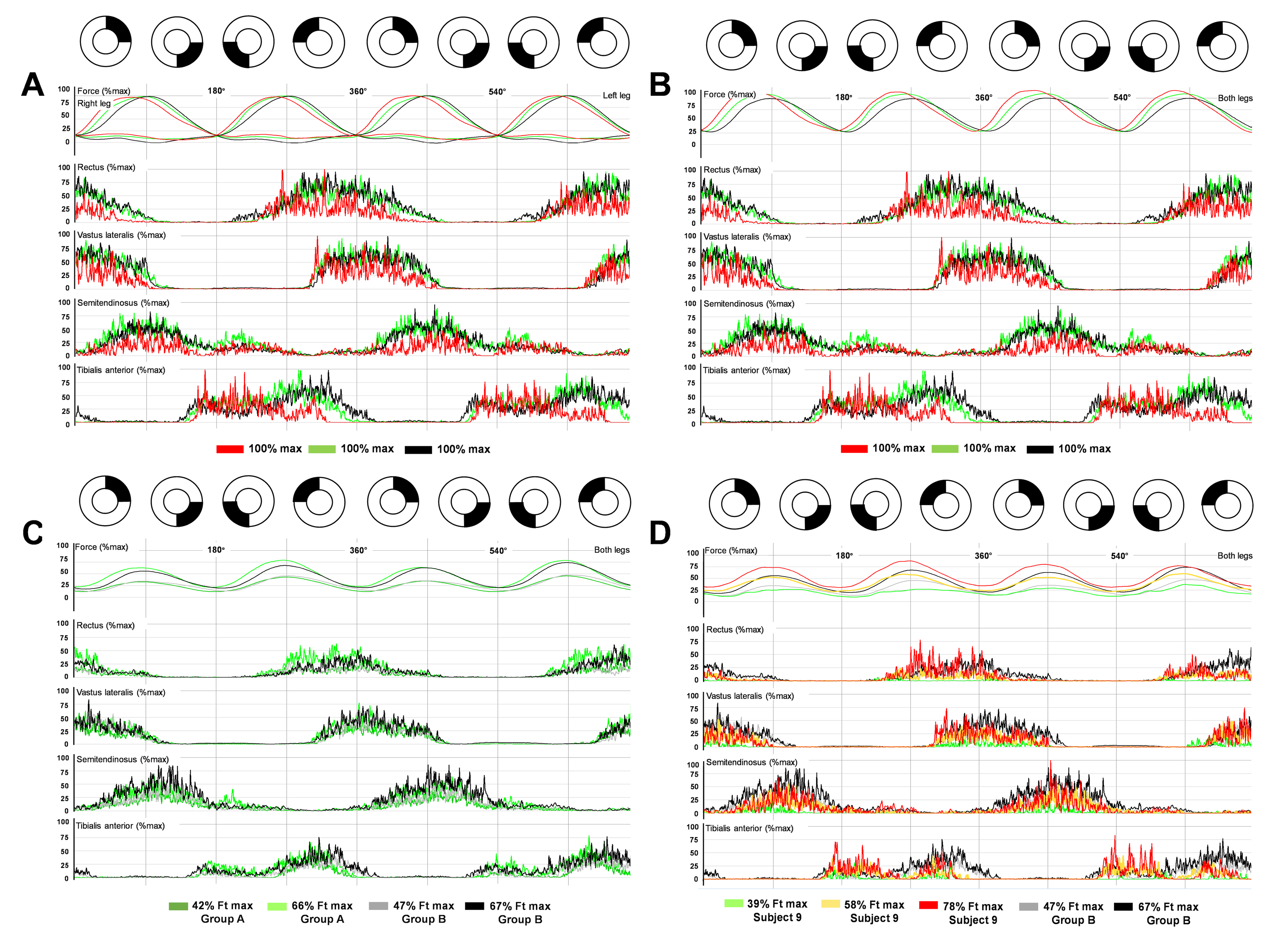

Supplement: S7 Fig — (A-D) Averaged tangential force and EMG data across two pedal cycles (0-720°) at different workloads (MP-tests) for subject 9 and group A/B (both n = 7). Pedaling technique clearly differs between subjects/groups in the maximum force/workload range (A, B), and at the transition from moderate to heavy domains of exercise (C, D). As with constant power (CP)-tests, we found stronger activation of the RF and VL in highly trained athletes, which reduces negative force (Fneg) in the second half of the pedal cycle (A), in favor of total propulsive force (Ftmax r+l) (B). This obviously advantageous muscle coordination pattern at the MLSSw also becomes apparent by comparison of group A vs group B (C) and subject 9 vs group B (D). For detailed description of data analysis and parameter definitions see Methods and S1 Fig. (TIF) [file pone.0282391.s007.tif]

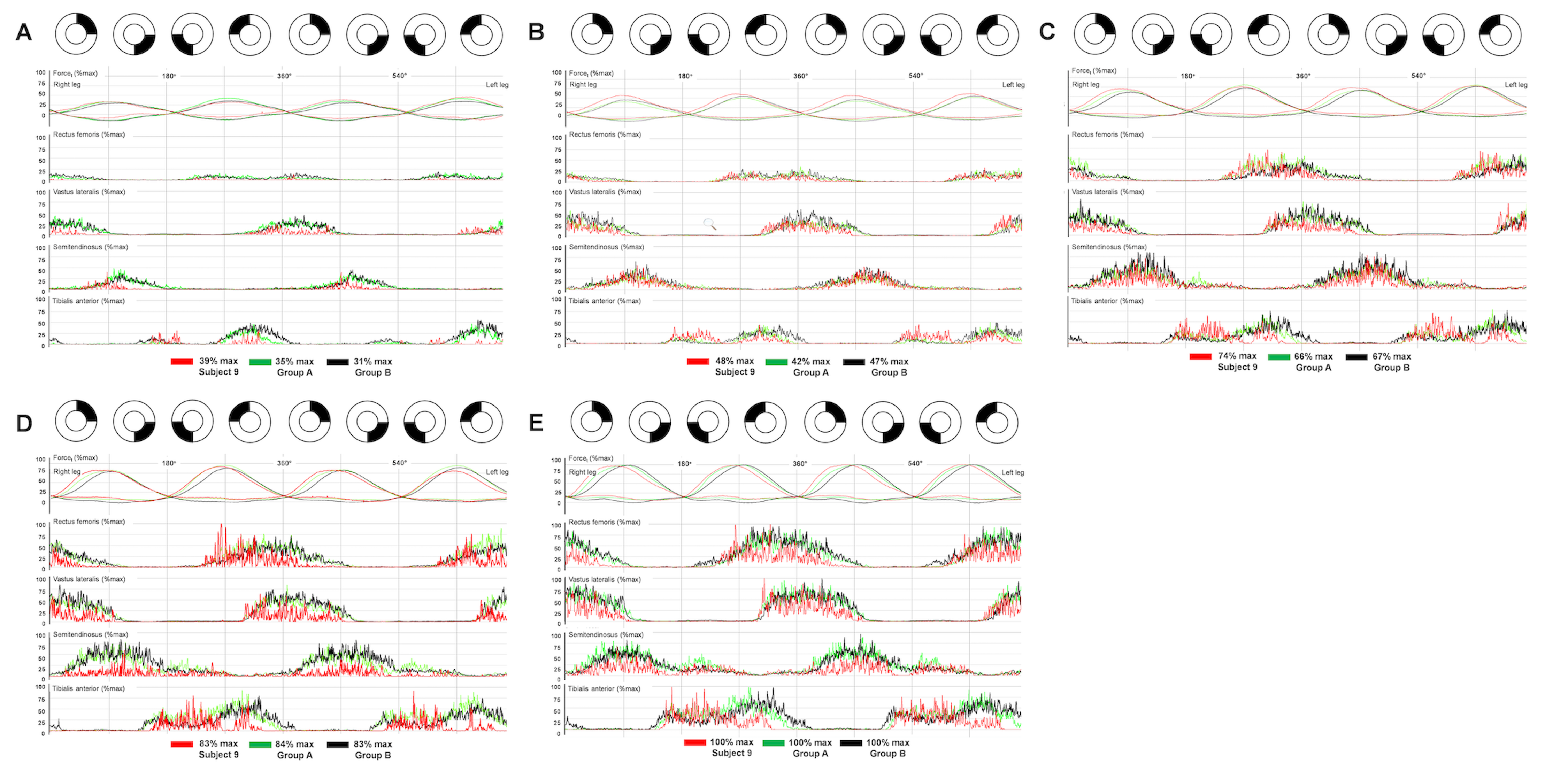

Supplement: S8 Fig — (A-E) Averaged tangential force and EMG data across two pedal cycles (0-720°) for five increments in MP-tests. Group A (green), group B (black) and subject 9 (red). For detailed description of data analysis and parameter definitions see Methods and S1 Fig. (TIF) [file pone.0282391.s008.tif]

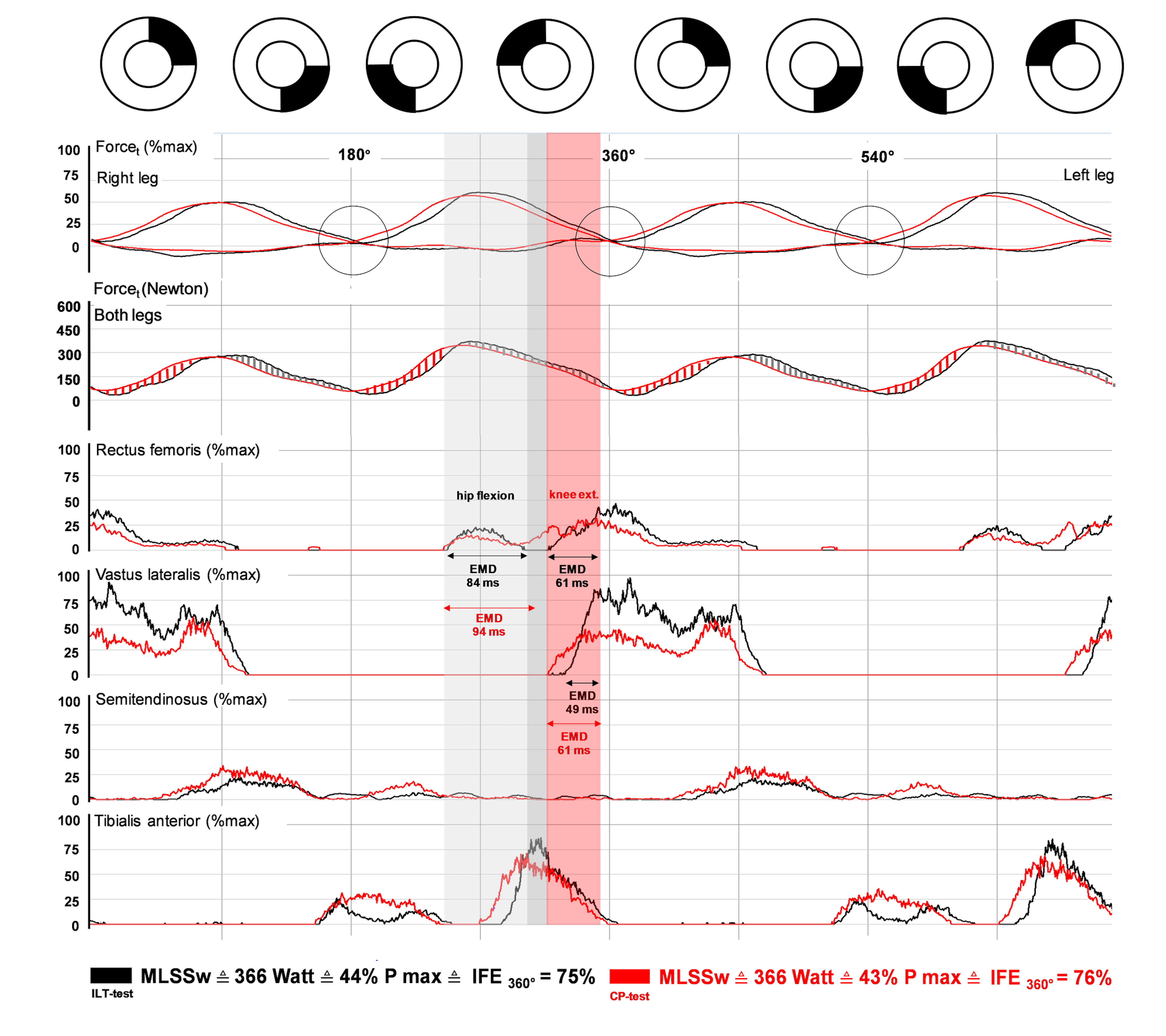

Supplement: S9 Fig — Averaged normalized bi-pedal crank force and EMG data across two pedal cycles (0–720°) at the MLSSw for ILT-tests (black) vs CP-tests (red) of subject 11 (top row). Second line depicts curves of absolute propulsive force (Newton). Note the longer delays of force crossings (DFCs), the different electromechanical delays (EMDs) for RF and VL and the shift in force development from Q1 to Q2 in the ILT-test. (TIF) [file pone.0282391.s009.tif]

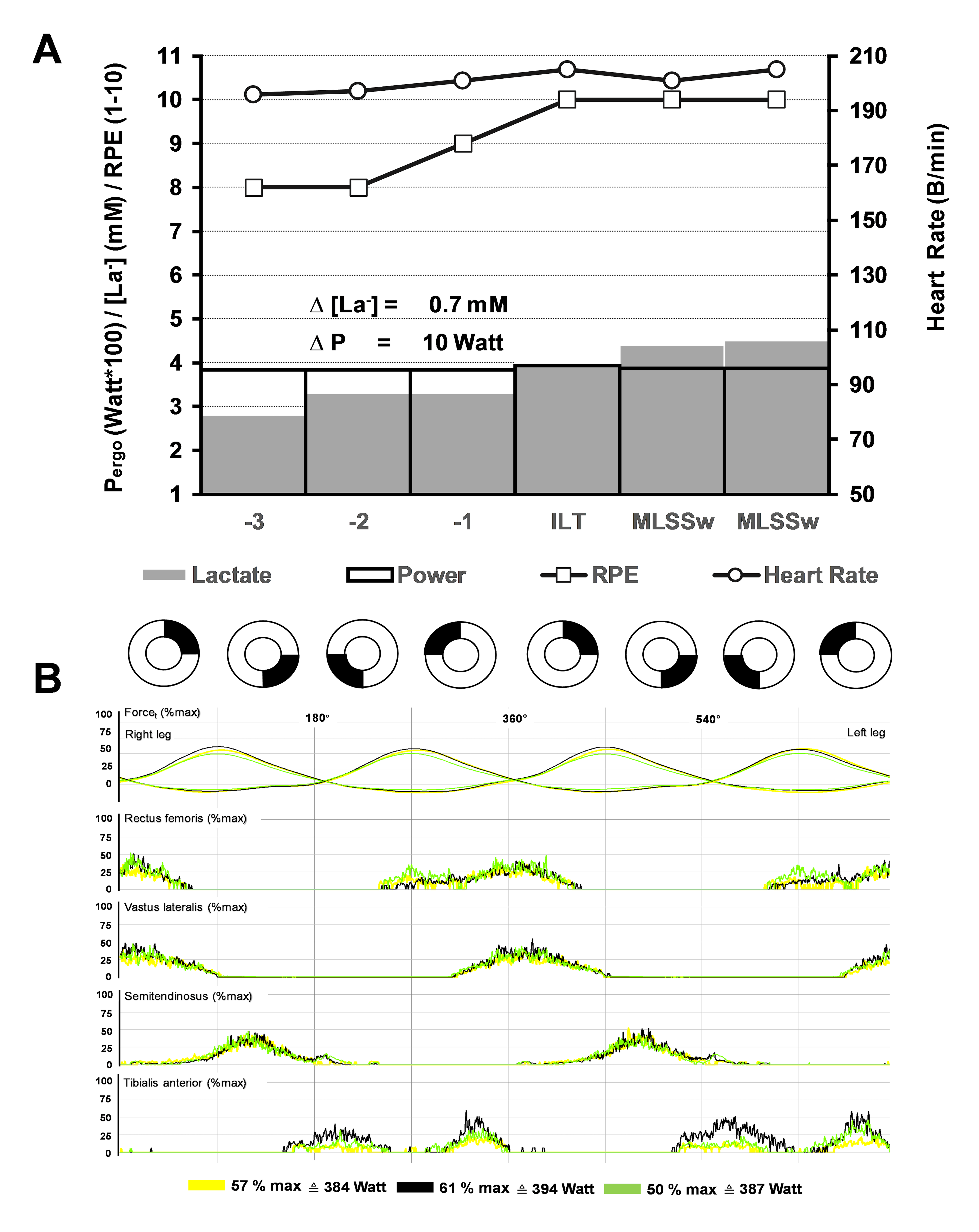

Supplement: S10 Fig — (A) Blood lactate concentration (mM), Power (Watt), rate of perceived exertion (RPE, 1-10) and heart rate (beats/min) for the final 6 increments of the individual lactate threshold (ILT)-test. A step-like blood lactate accumulation occurs at the individual lactate threshold (ILT, Δ[La−] = 0.7 mM) after a slight workload increment (ΔP = 10 Watt). Immediately after a comparable slight workload reduction (ΔP = 7-10 Watt), lactate accumulation slows down, indicating the maximal lactate steady state workload (MLSSw) [24]. (B) Averaged tangential force and EMG data across two pedal cycles (0–720°) for the pre-ILT stage, at the ILT and at the MLSSw. Note the elevated EMG activity of the TA at the ILT. One increment later (MLSSw), TA activity and negative tangential force (Fneg) are reduced, while early RF activation is enhanced. For detailed description of data analysis and parameter definitions see Methods and S1 Fig. (TIF) [file pone.0282391.s010.tif]
